# C646 inhibits G2/M cell cycle-related proteins and potentiates anti-tumor effects in pancreatic cancer

**DOI:** 10.1038/s41598-021-89530-8

**Published:** 2021-05-12

**Authors:** Hiroaki Ono, Tomotaka Kato, Yoshiki Murase, Yutaro Nakamura, Yoshiya Ishikawa, Shuichi Watanabe, Keiichi Akahoshi, Toshiro Ogura, Kosuke Ogawa, Daisuke Ban, Atsushi Kudo, Yoshimitsu Akiyama, Shinji Tanaka, Hiromichi Ito, Minoru Tanabe

**Affiliations:** 1 Department of Hepatobiliary and Pancreatic Surgery, Graduate School of Medicine, Tokyo Medical and Dental University, 1-5-45 Yushima, Bunkyo-ku, Tokyo, 113-8519 Japan; 2 Department of Surgery, College of Human Medicine, Michigan State University, Lansing, MI USA; 3 Division of Molecular Oncology, Graduate School of Medicine, Tokyo Medical and Dental University, Tokyo, Japan

**Keywords:** Surgical oncology, Pancreatic cancer

## Abstract

The activity of histone acetyltransferases (HATs) plays a central role in an epigenetic modification in cooperation with HDACs (histone deacetyl transferases). It is likely that malfunction of this enzymatic machinery controlling epigenetic modification is relevant to carcinogenesis and tumor progression. However, in pancreatic cancer, the clinical relevance of HAT activity and histone acetylation has remained unclear. We identified that H3 acetylation was expressed in all pancreatic cancer patients, indicating that H3 acetylation may be essential in pancreatic cancer cells. We also found that the HAT inhibitor C646 augmented anti-tumor effects in vitro by inhibiting cell proliferation and cell cycle progression concomitantly with suppression of acetylated H3K9 and H3K27 expression. C646 or p300 and CBP (CREB-binding protein)-specific siRNA treatment inhibited the transcription of the G2/M cell cycle regulatory proteins cyclin B1 and CDK1 (cyclin-dependent kinase 1). C646 treatment also inhibited tumor growth in vivo in a xenograft mouse model. C646 could be an effective therapeutic agent for pancreatic cancer. The epigenetic status of pancreatic cancers based on their level of histone H3 acetylation may influence patient survival. Epigenetic stratification according to H3K27 acetylation could be useful for predicting disease prognosis as well as the therapeutic efficacy of C646 in pancreatic cancer.

## Introduction

Pancreatic cancer remains a fatal disease due to its malignant phenotype and high relapse rate even though recent advances in medical technology for early diagnosis and treatment of many other cancers have engendered markedly improved outcomes. The opposite is unfortunately the case for pancreatic cancer, the morbidity and mortality rate of which continues to increase to such a degree that it is predicted to become the second leading cause of cancer death within a decade in the United States^1,2^.

To characterize common mechanisms of oncogenesis in pancreatic cancer, earlier work focused on genetic alterations and had defined gene mutations such as activation of KRAS and inactivation of TP53, DPC4, or CDKN2A that were crucial for carcinogenesis^3,4^. Subsequently, four different disease subgroups have been proposed according to a comprehensive molecular examination of DNA mutations and mRNA expression analysis; these subtypes are recognized as invaluable for the selection of therapeutic interventions and for the prediction of clinical outcomes^5^. Recently, in addition to these transcriptional subtypes, it has been established that epigenetic mechanisms contribute to tumor progression^6^.

Epigenetic modification such as histone acetylation and histone deacetylation plays a central role in producing divergent gene structures influencing functional phenotypes. Epigenetic pathways are tightly regulated by the opposing functions of HATs (histone acetyltransferases) and HDACs (histone deacetyl transferases). Histone acetylation is associated with the transcriptionally active form of chromatin and is characterized by acetylation of histone H3 on Lys9 and Lys27, which serve as a marker of active enhancement^7,8^. In contrast, histone deacetylation reflects a condensed and transcriptionally silent chromatin structure. Accordingly, it is believed that an imbalance in the activation of these enzymes can contribute to malignant transformation and tumor progression^6,9^.

HAT activity is correlated with gene transcriptional programs involved in fundamental cellular processes such as DNA replication and cell cycle progression^10,11^. For this reason, it has been proposed that inhibition of HAT activity could represent a potential therapeutic target in different types of cancer^12–17^. However, compared with the data available for HDAC, the biological contribution of histone acetylation to cancer progression in pancreatic cancer seems to have been under investigated thus far^6^. Recently, the small molecule HAT inhibitor C646 has been shown by in silico screening approach to exert a selective inhibitory action against p300 and CBP (CREB-binding protein)^17^. It was demonstrated that C646 attenuated histone acetylation through p300/CBP blockade and impeded cancer proliferation in some cancer cell lines.

The underlying mechanism of HAT activity and histone acetylation has not been established in pancreatic cancer. In the present study, we sought to evaluate histone H3 acetylation status in surgically resected samples of pancreatic cancer ex vivo, investigate the effect of in vitro HAT inhibition on cell proliferation and cell cycle distribution using the HAT inhibitor C646, and evaluate the inhibitory efficacy of C646 on tumor growth in vivo. Our study sheds light on the phenomena affected by HAT inhibition in pancreatic cancer cells.

## Results

### C646 inhibits histone H3 acetylation and proliferation of pancreatic cancer cells

**Figure 1:**
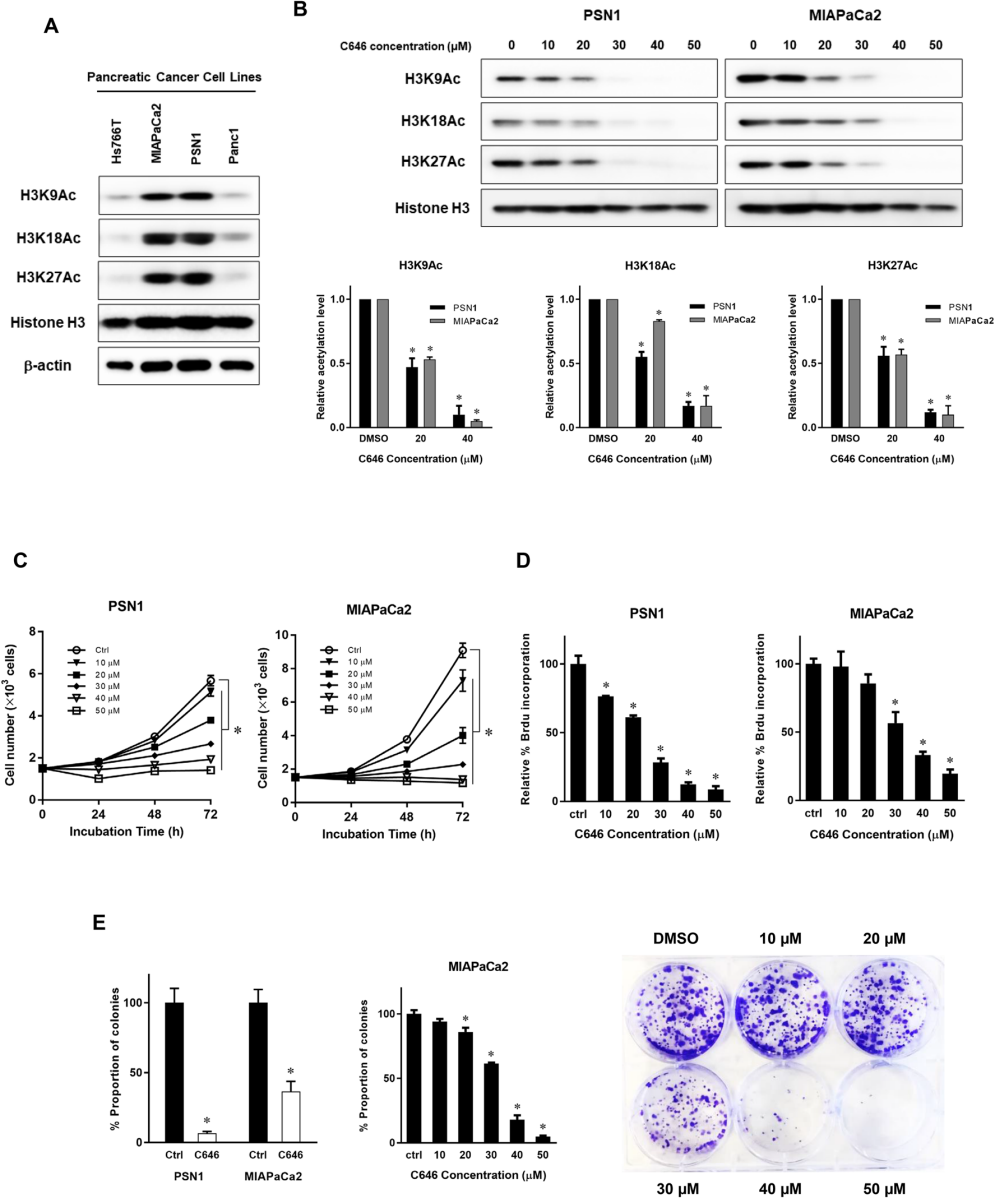
C646 inhibits histone H3 acetylation and proliferation of pancreatic cancer cells. (**A**) Endogenous H3K9Ac, H3K18Ac, and H3K27Ac expression in human pancreatic cancer cell lines. Histone H3 acetylation levels were quantified in four pancreatic cancer cell lines, Hs766T, MIAPaCa2, PSN1, and Panc1, by Western blotting. (**B**) C646 treatment (10–50 µM) compared with the DMSO vehicle control by Western blotting of PSN1 and MIAPaCa2 cells. Histone H3K9, H3K18, and H3K27 acetylation were downregulated as the C646 concentration increased. Experiments were performed in duplicate. Error bars represent mean ± SD. *p < 0.05. (**C**, **D**) Cell viability following C646 treatment of PSN1 and MIAPaCa2 cells by WST-8 assay (**C**) and BrdU incorporation assay (**D**). (**C**) Cells were seeded at 1.5 × 10^3^ per well and incubated overnight, after which titrated doses of C646 (10–50 µM) were added. Cell viability assays were performed every 24 h up to 72 h. (**D**) C646 treatment was for 72 h. Each data point was evaluated as relative % ratio normalized to vehicle control. Each experiment was performed in duplicate. Error bars represent mean ± SD. *p < 0.05 by one-way ANOVA with post hoc Dunnett's test. (**E**) Left panel, clonogenic cell survival after C646 treatment relative to DMSO vehicle control for PSN1 and MIAPaCa2 cells. Cancer cells were treated with 30 µM C646 or DMSO for nine hours and then incubated for seven days in fresh media. The ability to form colonies after C646 treatment was significantly decreased in both cell lines. *p < 0.05 vs control. Middle panel, dose-dependent effects on the clonogenic assay for MIAPaCa2 cells (*p < 0.05 vs control by one-way ANOVA with post hoc Dunnett's test). Right panel, representative image of clonogenic cell survival assay on treatment of MIAPaCa2 cells with different doses of C646. Error bars represent mean ± SD.

H3K9Ac, H3K18Ac, and H3K27Ac expression was first quantified by Western blotting in four established human pancreatic cancer cell lines (Hs766T, MIAPaCa2, PSN1, and Panc1). This revealed that all four cell lines manifested histone H3 acetylation, but at different levels (Fig. 1A). In vitro experiments using the HAT inhibitor C646 was performed to evaluate any functional implications of histone H3 acetylation for pancreatic cancer proliferation. Histone H3 acetylation in MIAPaCa2 and PSN1 cells was effectively inhibited by 20–30 µM of C646 (Fig. 1B). In Panc1 cells, which have a lower expression of histone acetylation, it was slightly inhibited by 40 µM of C646 (Supplementary Figure S1A). Cell proliferation, DNA synthesis, and clonogenic growth were also reduced in step with increased inhibition of histone acetylation as the C646 dose increased (Fig. 1C–E and Supplementary Figure S1B). These results strongly indicated that a positive correlation exists between histone H3 acetylation levels and the inhibitory efficacy of C646 treatment on cell growth of pancreatic cancer cell lines.

### C646 induces G2/M arrest and apoptosis through regulation of cell cycle-associated genes

**Figure 2:**
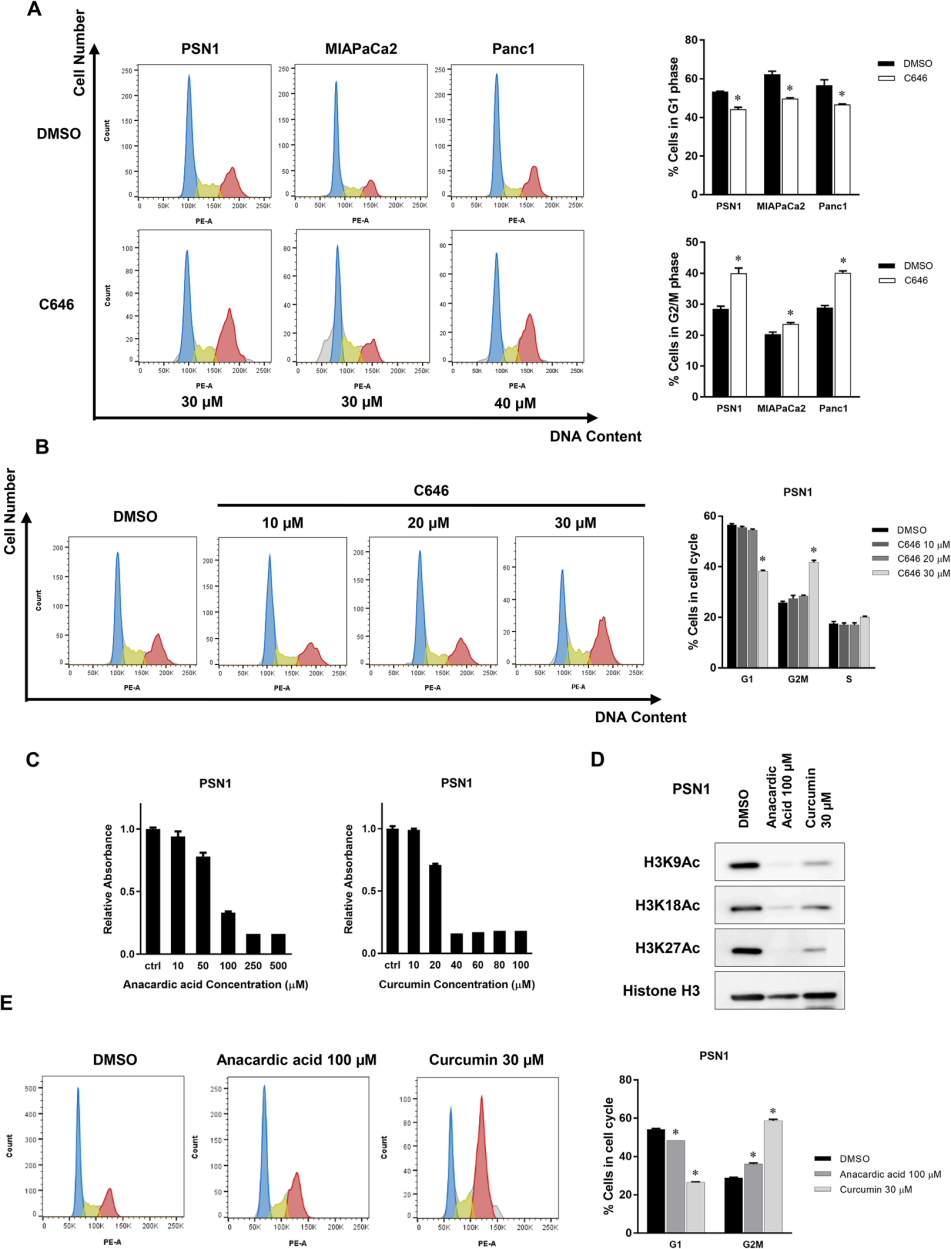
C646 induces G2/M arrest in pancreatic cancer cells. (**A**) Effects of C646 treatment on cell cycle progression. Flow cytometry was performed after 30 µM C646 treatment in PSN1 and MIAPaCa2 cells and 40 µM C646 in Panc1 cells for 48 h. C646 treatment decreased the number of cells arresting in G1 phase and increased cells accumulating in G2/M in all three pancreatic cancer cell lines. Error bars represent mean ± SD. *p < 0.05 vs DMSO vehicle control. (**B**) Dose-dependency of C646-induced G2/M cell cycle arrest in PSN1 cells. Error bars represent mean ± SD. *p < 0.05 vs DMSO vehicle control. (**C**) Cell viability following treatment with the HAT inhibitors curcumin and anacardic acid for 72 h and analysis of dose dependence by WST-8 assay in PSN1 cells. Error bars represent mean ± SD. (**D**) Effects of curcumin and anacardic acid on histone H3 acetylation. Acetylation of H3K9, H3K18, and H3K27 were assessed following 72-h treatment of curcumin and anacardic acid. Histone H3 acetylation of H3K9, H3K18, and H3K27 were effectively downregulated by curcumin (30 µM) and anacardic acid (100 µM). (**D**) Effects of curcumin (30 µM) and anacardic acid (100 µM) on cell cycle progression following 48-h incubation in PSN1 cells. Notably, curcumin and anacardic acid induced G2/M cell cycle arrest in PSN1 cells. Error bars represent mean ± SD. *p < 0.05 vs DMSO vehicle control.

The effect of inhibition of histone H3 acetylation on the distribution of cells at different phases of the cell cycle after C646 treatment was subsequently investigated. Inhibition of histone H3 acetylation significantly decreased the proportion of cells in G1 and increased the proportion in G2/M in three pancreatic cancer cell lines (Fig. 2A). The induction of G2/M arrest paralleled the effectiveness of inhibition of H3 acetylation and the dose of C646 in PSN1 cells (Fig. 2B).

Subsequently, we assessed the effect of other HAT inhibitors on the G2/M cell cycle distribution to determine whether they had an effect similar to that of C646 treatment. In addition to C646, curcumin and anacardic acid have also been characterized as HAT inhibitors^18,19^. Those drugs inhibited H3 acetylation and induced G2/M arrest at the same concentration at which proliferation of cancer cells were effectively inhibited (30 µM for curcumin and 100 µM for anacardic acid) (Fig. 2C–E).

**Figure 3:**
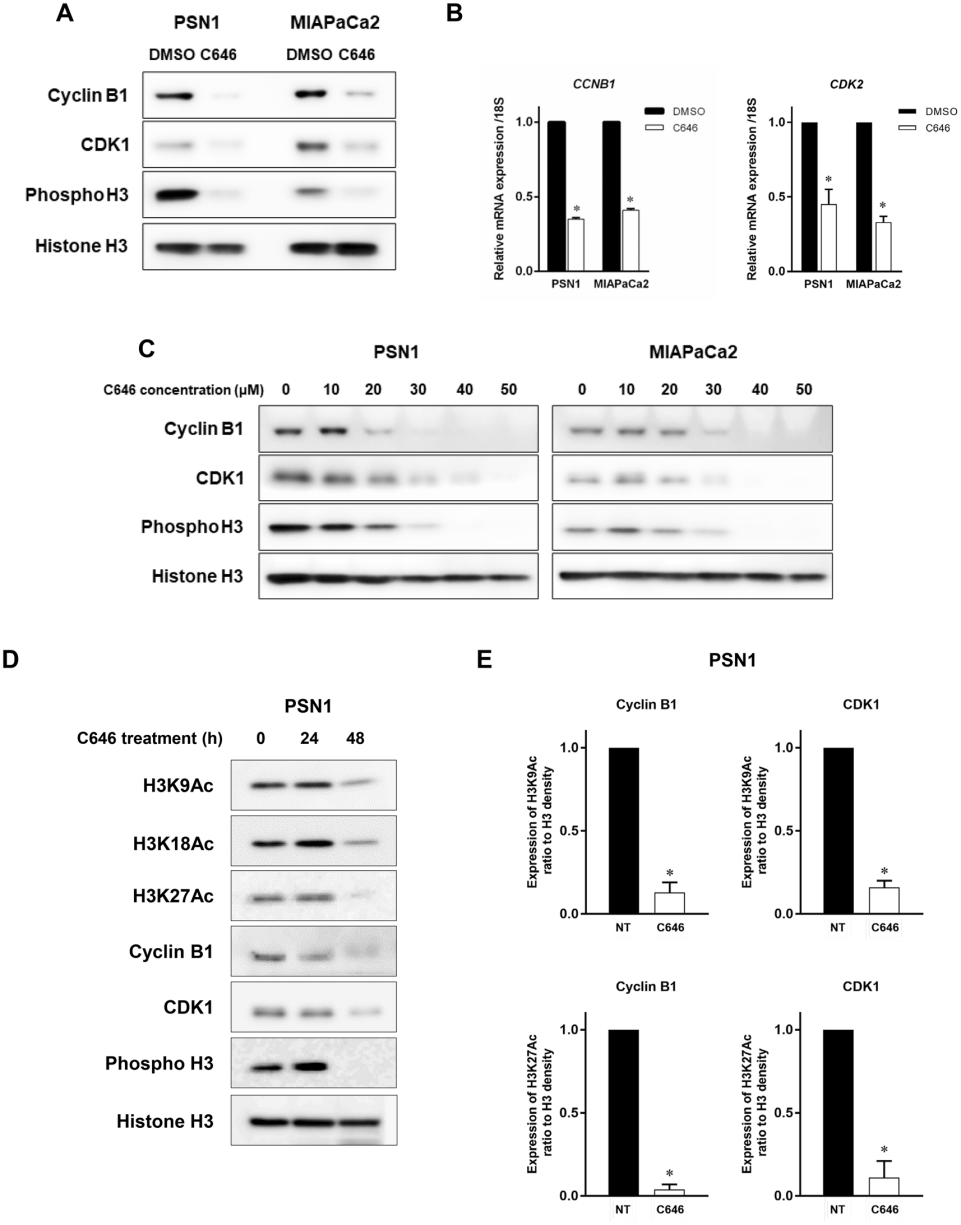
C646 inhibits expressions of G2/M cell cycle-associated genes. (**A**, **B**) Expression profiles of molecules associated with the G2/M transition at the protein level (**A**) and mRNA level (**B**) following C646 treatment. mRNA expression of cyclin B1 and CDK1 was inhibited after 48-h treatment with 30 µM C646 treatment in PSN1 and MIAPaCa2 cells. Inhibition of protein expression of cyclin B1 and CDK1 after 72-h C646 treatment was confirmed by Western blotting. Phosphorylated histone H3 (Ser10), recognized as an M phase marker, was also inhibited by C646 treatment. Error bars represent mean ± SD. *p < 0.05 vs DMSO vehicle control. (**C**) Dose-dependent analysis of G2/M cell cycle-associated molecules following C646 treatment (10–50 µM) by Western blotting in PSN1 and MIAPaCa2 cells. (**D**) Time-dependent analysis of histone H3 acetylation and G2/M cell cycle-associated molecules following C646 treatment for 24 and 48 h in PSN1 cells. (**E**) Quantitative ChIP analysis of cyclin B1 and CDK1 with C646 treatment (40 µM) in PSN1 cells. H3K9ac and H3K27ac levels at the promoter region of cyclin B1 and CDK1 were significantly decreased by C646 treatment. Error bars represent mean ± SD. *p < 0.05 vs controls.

C646 downregulated phosphorylation of H3 (Ser10), which is known to be associated with chromatin condensation in the late G2 and M phases, and is recognized as a specific mitotic marker^20^. Cell cycle-associated proteins such as cyclin B1 and CDK1 function as key molecules during G2/M entry^21^, and C646 treatment was found to inhibit cyclin B1 and CDK1 expression at the protein and mRNA level (Fig. 3A–C). Inhibition of these G2/M cell cycle-associated proteins is regulated at the transcriptional level. The suppression of histone acetylation and cell cycle-associated gene expression of cyclin B1 and CDK1 became prominent 48 h after the start of C646 treatment (Fig. 3D). Even in Panc1 cells with low H3 acetylation, phospho-H3, cyclin B1, and CDK1 were also reduced by C646 treatment (Supplementary Figure S1C). We next investigated whether cyclin B1 and CDK1 expression are regulated by histone H3 acetylation. When treated with C646, the level of histone acetylation of cyclin B1 and CDK1 promotor was significantly inhibited in PSN1 cells (Fig. 3E). The transcription of cyclin B1 and CDK1 was regulated by H3K9 and H3K27 acetylation, and molecules associated with G2/M entry were likely direct functional targets regulated by histone acetylation.

**Figure 4:**
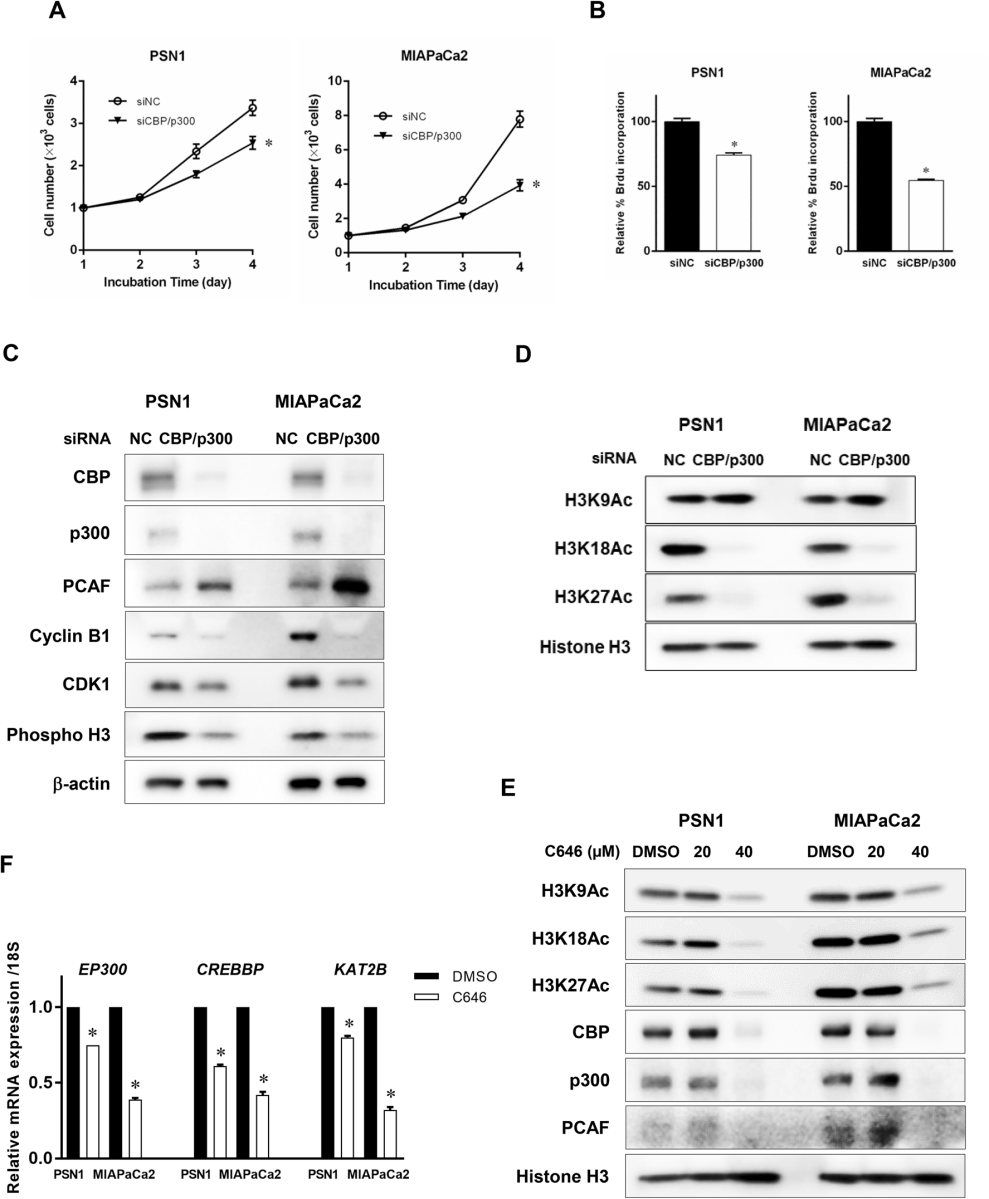
P300 and CBP dual knockdown inhibits CDK1 and cyclin B1 expression and H3K18 and H3K27 acetylation, but not H3K9 acetylation. (**A**, **B**) Cell viability following CBP and p300 siRNA treatment as determined by WST-8 assay (**A**) and BrdU incorporation assay (**B**). Cells were transfected with CBP- and p300-specific siRNAs or negative control siRNA for 48 h and cell viability was assessed every 24 h through 72 h (WST-8 assay) (**A**) or at 72 h (BrdU incorporation assay) (**B**). Error bars represent mean ± SD. *p < 0.05 vs controls treated with negative control siRNA by one-way ANOVA with post hoc Dunnett's test (**A**) and t-test (**B**). (**C**) Effects on CBP, p300, and PCAF expression and expression of G2/M cell cycle regulatory molecules after p300 and CBP siRNA treatment. Effective knockdown of CBP and p300 by treatment with CBP- and p300-specific siRNAs was confirmed at the protein level. PCAF expression was increased by p300 and CBP gene silencing. Expression of G2/M cell cycle regulatory molecules were suppressed. Cancer cells were treated with CBP- and p300-specific siRNAs or negative control siRNA for 72 h. (**D**) Effects of silencing both p300 and CBP genes on histone H3 acetylation. Effects on acetylation of H3K9, H3K18, and H3K27 were assessed. Acetylated H3K18 (H3K18Ac) and H3K27 (H3K27Ac) were downregulated by CBP and p300 gene silencing, while acetylated H3K9 (H3K9Ac) was not affected. (**E**) Effects of C646 treatment on histone H3 acetylation and CBP, p300, and PCAF expression in PSN1 and MIAPaCa2 cells. Cancer cells were treated with C646 for 72 h at each concentration (20 and 40 µM). At a C646 concentration of 40 µM, which sufficiently inhibited histone acetylation, expression of these HAT molecules was downregulated. (**F**) Effects of CBP, p300, and PCAF mRNA levels following C646 treatment. mRNA expression of p300, CBP, and PCAF were inhibited after 48-h treatment with 30 µM C646 treatment in PSN1 and MIAPaCa2 cells.

It has been reported that CBP and p300 are required for efficient H3K27 acetylation^22^. We previously noted that p300 knockdown downregulates H3K27 acetylation^23^. Here, we confirmed that CBP and p300 siRNA-mediated treatment evoked a response similar to that of C646 treatment. Certainly, CBP and p300 double-knockdown inhibited proliferation of pancreatic cancer cells as C646 treatment (Fig. 4A, B). Furthermore, CBP and p300 double-knockdown downregulated cyclin B1 and CDK1 expression at both the protein and transcriptional level (Fig. 4C and Supplementary Figure S2). Interestingly, knocking down both CBP and p300 also suppressed acetylated H3K18 and H3K27, whereas acetylated H3K9 was not affected by CBP and p300 gene silencing (Fig. 4D).

H3K9 acetylation is distinctively regulated by PCAF expression, whereas H3K18 and H3K27 acetylation is mediated by p300 and CBP expression^24^. It was suggested that the difference in H3K9 acetylation between these models is attributable to the distinct role of PCAF expression. C646 treatment decreased the expression of the HAT molecule PCAF identically to that of p300 and CBP, indicating that the effect of histone acetylation of C646 functions to inhibit HAT activity more broadly than anticipated (Fig. 4E). Alterations in the expression of CBP, p300, and PCAF in response to C646 was regulated by the transcriptional levels. In fact, mRNA expression of these genes was reduced by C646 treatment in PSN1 and MIAPaCa2 cells as shown in Fig. 4F. On the other hand, the effect of both p300 and CBP gene silencing on histone acetylation seems to be limited in comparison to C646 treatment. As shown in Fig. 4C, PCAF expression was markedly increased in cancer cells with p300 and CBP double knockdown. It is probable that PCAF expression is upregulated in a manner reciprocal to that of p300 and CBP gene silencing.

**Figure 5:**
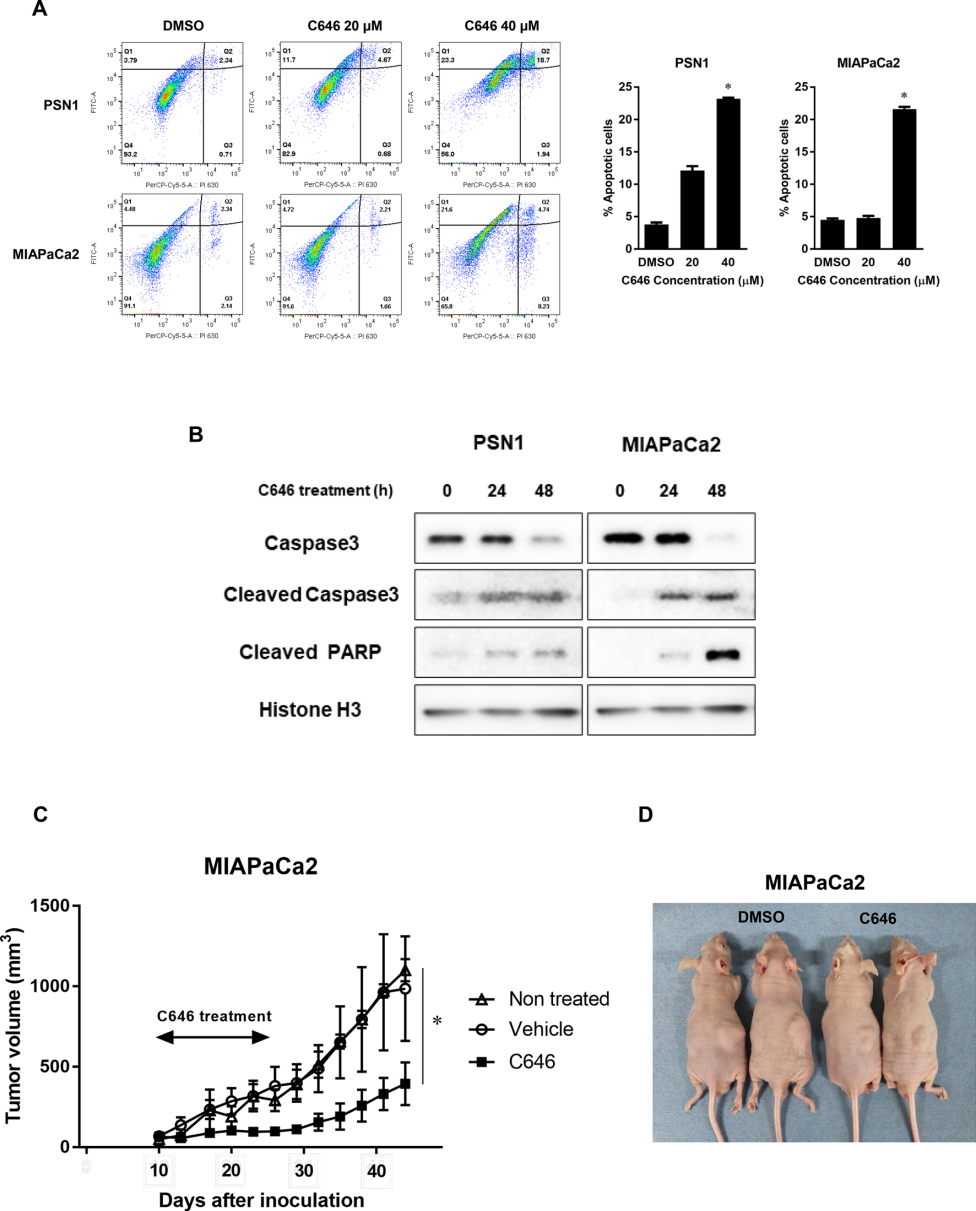
C646 induces apoptosis of pancreatic cancer cells. Effects of C646 on apoptosis (**A**)**/**(**B**) and therapeutic efficacy of C646 treatment against pancreatic tumors transplanted in nude mice (**C**)**/**(**D**). (**A**) Cancer cells were treated with C646 at 30 µM for 48 h and fixed in 70% ethanol at − 20 °C overnight. Fixed cells were stained with for annexin V-FITC and propidium iodide. The proportions of apoptotic cells were significantly increased as C646 concentration was increased, compared with the control cells. Error bars represent mean ± SD. *p < 0.05 vs controls. (**B**) Cancer cells were treated with C646 at 30 µM for 48 h. The expression of apoptotic markers was increased by C646 treatment compared with controls. (**C**) Mice were inoculated with MIAPaCa2 cells in the flank by subcutaneous injection. Once tumors reached measurable size in 10 days, C646 (10 mg/kg) or vehicle (DMSO) were administrated by i.p. injection daily for two weeks. The dose of C646 was set to 10 mg/kg, which is the lower dose in this study, referring to previous literature^47^. The volume of the tumors in mice was recorded over for eight weeks (C646, vehicle, and nontreated; each N = 4). C646 treatment in pancreatic cancer suppressed the growth of the tumor significantly (*p < 0.01 by one-way ANOVA with post hoc Dunnett's test). Error bars represent mean ± SD. (**D**) Representative images of transplanted tumors in mice at the time of necropsy (left panel, vehicle; right panel, C646).

Inhibition of histone H3 acetylation also resulted in inhibition of proliferation and induction of apoptosis, which increased in parallel with the degree of inhibition of histone H3 acetylation (Fig. 5A, B). Hence, complete inhibition of histone acetylation resulted in a high degree of apoptosis induction in pancreatic cancer cells. We then evaluated the therapeutic efficacy of inhibition of histone acetylation by C646 treatment in mouse xenografts in vivo*.* Tumor growth was suppressed during 2-week treatment with C646. We demonstrated that C646 treatment significantly decreased cancer cell growth in tumor-bearing nude mice (985.5 mm^3^ vs. 394.7 mm^3^ for tumors in MIAPaCa2 cells, *p* < 0.01; Fig. 5C, D).

### An intermediate level of expression of H3K27Ac is associated with poorer prognosis in pancreatic cancer

**Figure 6:**
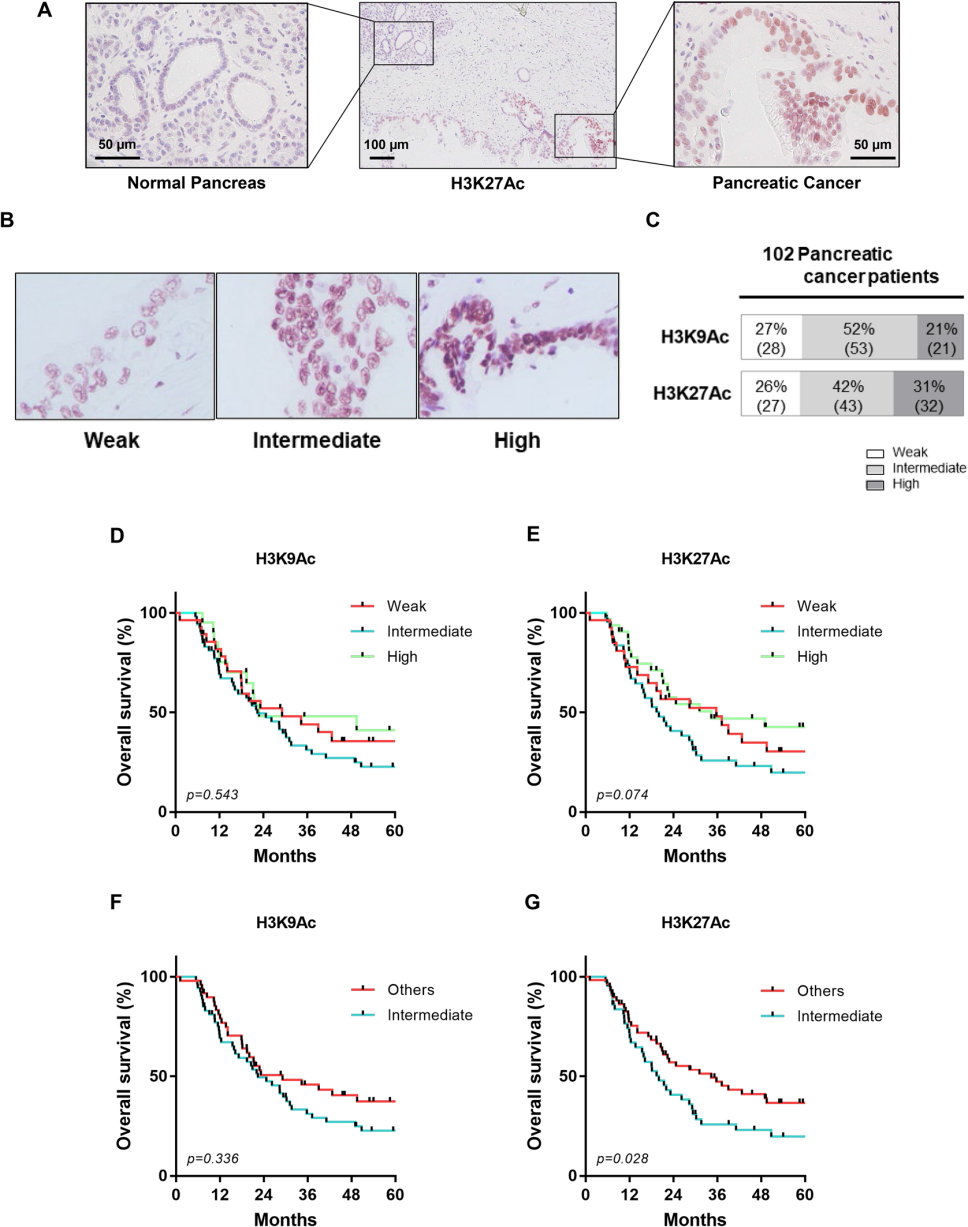
Clinical significance of histone acetylation in pancreatic cancer cells. (**A**) Representative immunohistochemical staining of pancreatic cancer with positive H3K27Ac expression concomitant with normal pancreas tissue. Left panel: adjacent normal pancreatic tissue. Right panel: pancreatic cancer. (**B**) Representative immunohistochemical images of weak, intermediate, and high expression of histone acetylation in pancreatic cancer tissues. For scoring H3K9 and H3K27 acetylation, the intensity was graded using high-power (× 200) microscopy and evaluated by the intensity randomly chosen 3 fields from each specimen. Histone H3 acetylation of K9 and K27 staining were scored for nuclear intensity with numerical scores of 1, 2 and 3 representing weak, intermediate, and high staining intensity. Expression of histone acetylation can also be identified by staining patterns in the nucleus of cancer cells. Speckled low staining pattern with weak expression and diffuse strong staining pattern with high expression were observed. Intermediate expression showed mixed moderate staining pattern with speckled and diffuse staining. If the staining was homogenous within a given tumor, it was evaluated by intensity scores. If the staining is heterogeneous, then the assigned score was that observed in ≥ 50% or the majority of the nuclear of the cancer cells. (**C**) The frequency of pancreatic cancer patients with weak, intermediate, or high staining for H3K9Ac and H3K27Ac. Of note, all pancreatic cancer samples were positive for H3K9aAc and H3K27Ac expression to some degree. (**D**–**G**) Kaplan–Meier curves for survival rates (overall survival) of pancreatic cancer patients according to H3K9Ac and H3K27Ac expression. (**D**), (**F**) H3K9Ac expression. (**E**), (**G**) H3K27Ac expression. Subgroup analysis was performed stratifying patients into intermediate acetylated H3 expression-vs-pooled weak and high acetylated H3 expression. Intermediate H3K27Ac immunoreactivity in tumor cells was significantly associated with worse survival (*p* = 0.028).

Finally, we examined ex vivo histone H3 acetylation using H3K9Ac and H3K27Ac immunohistochemical staining of tumor tissues to investigate the biological role of histone H3 acetylation in pancreatic cancer. A total of 102 pancreatic cancer patients were evaluated in this study. The clinical characteristics of the patients were shown in Table 1. It was notable that all pancreatic cancer samples were positive for H3 acetylation, albeit to quite different degrees. Overall, histone acetylation was increased more in pancreatic cancer tissues than in normal pancreas tissues as indicated by immunohistochemical staining (Fig. 6A and Supplementary Figure S3). We next stratified the histone acetylation levels of cancer cells into three staining patterns as weak, intermediate, and high histone acetylation (Fig. 6B). Intermediate expression of H3K9Ac and H3K27Ac was observed more frequently than either hypoacetylation (weak histone H3 acetylation) or hyperacetylation (high histone H3 acetylation). As shown in Fig. 6C, 52% of patients showed intermediate expression of H3K9Ac and 42% for H3K27Ac. Nonetheless, there was no linear relationship between H3K9Ac and H3K27Ac expression (Spearman r = 0.1026).

**Table 1:** Patient characteristics.

	H3K9Ac				H3K27AC			
	Weak	Intermediate	High	*P* value	Weak	Intermediate	High	*P* value
**Age, years (mean ± SD)**	72.0 ± 7.6	68.7 ± 8.5	67.3 ± 10.4	0.110	68.5 ± 6.7	69.7 ± 10.1	69.4 ± 8.5	0.858
**Gender**				0.707				0.772
Male	19 (68%)	35 (66%)	12 (57%)		19 (70%)	27 (63%)	20 (63%)	
Female	9 (32%)	13 (34%)	9 (43%)		8 (30%)	16 (37%)	12 (37%)	
**CEA, ng/ml (mean ± SD)**	8.7 ± 20.6	6.1 ± 8.2	3.0 ± 2.2		5.8 ± 8.8	7.4 ± 17.4	4.7 ± 4.1	0.637
**CA19-9, U/ml (mean ± SD)**	625 ± 1621	358 ± 891	147 ± 299		352 ± 619	488 ± 1490	288 ± 654	0.723
**Tumor location**				0.264				0.209
Head	21 (75%)	30 (57%)	13 (62%)		14 (52%)	31 (72%)	19 (59%)	
Body/tail	7 (25%)	23 (43%)	8 (38%)		13 (48%)	12 (28%)	13 (41%)	
**Extrapancreatic invasion**				0.713				0.153
Positive	25 (89%)	45 (85%)	17 (81%)		20 (74%)	38 (88%)	29 (91%)	
Negative	3 (11%)	8 (15%)	4 (19%)		7 (26%)	5 (12%)	3 (9%)	
**Portal Vein invasion**				0.800				0.494
Positive	7 (25%)	14 (26%)	4 (19%)		6 (22%)	13 (30%)	6 (23%)	
Negative	21 (75%)	39 (74%)	17 (81%)		21 (78%)	30 (70%)	26 (77%)	
**Venous invasion**				0.117				0.724
Positive	17 (61%)	43 (81%)	14 (67%)		18 (67%)	33 (77%)	23 (72%)	
Negative	11 (39%)	10 (19%)	7 (33%)		9 (33%)	10 (23%)	9 (28%)	
**Neural invasion**				0.742				0.052
Positive	20 (71%)	34 (64%)	13 (62%)		15 (56%)	34 (79%)	18 (56%)	
Negative	8 (29%)	19 (36%)	8 (38%)		12 (44%)	9 (21%)	14 (44%)	
**Lymphatic invasion**				0.094				0.389
Positive	18 (64%)	37 (70%)	9 (43%)		14 (52%)	29 (67%)	21 (66%)	
Negative	10 (36%)	16 (30%)	12 (57%)		13 (48%)	14 (33%)	11 (34%)	
**T status (AJCC7)**				0.809				0.566
pT1, < 20 mm	5 (18%)	7 (13%)	2 (10%)		6 (22%)	3 (7%)	5 (16%)	
pT2, 20–40 mm	14 (50%)	31 (60%)	11 (52%)		15 (56%)	24 (57%)	17 (53%)	
pT3, 40 mm ≤	9 (32%)	14 (27%)	8 (38%)		6 (22%)	15 (36%)	10 (31%)	
**N status (AJCC7)**				0.906				0.124
pN0	8 (29%)	15 (28%)	7 (33%)		10 (37%)	8 (15%)	12 (38%)	
pN1	20 (71%)	38 (72%)	14 (67%)		17 (63%1	35 (85%)	20 (62%)

In terms of the clinical significance of histone H3 acetylation in pancreatic cancer cells, hyperacetylation tended to be associated with a better prognosis for both H3K9Ac and H3K27Ac expression, whereas intermediate levels tended to be correlated with a worse prognosis (Fig. 6D, E). For H3K27Ac expression, not for H3K9Ac expression, this achieved significance, especially when intermediate histone H3 acetylation was compared with the two groups that showed either hypoacetylation or hyperacetylation (Fig. 6F, G). Here, the median overall survival (OS) was 34.4 months for intermediate expression versus 20 months for higher and lower pooled expression (log-rank test *p* = 0.028).

Univariate Cox regression analyses indicated that lymphatic invasion, venous invasion, N status, and H3K27Ac expression status were all significant risk factors for OS of pancreatic cancer patients. Multiple regression analysis for these four variables showed that venous invasion was an independent risk factor (*p* = 0.023; confidence interval (CI) 1.12–4.62) (Supplementary Table S1).

## Discussion

Transcriptional dysregulation is a fundamental characteristic of the cellular genetic and epigenetic landscapes in carcinogenesis^25,26^. Enhancers are DNA sequences that regulate transcription by binding to their respective transcription factors. Their activity is usually regulated by properties related to chromatin status in terms of epigenetic modifications such as histone acetylation or mono-methylation^8,27,28^. Dysregulation of the enzymatic machinery associated with histone modification often accompanies oncogenic transformation^29,30^. Recently, the notion that large clusters of transcriptional enhancers regulate gene alterations and affect cell identity and disease state has gained traction^31^. Such so-called “super-enhancers” are also involved in oncogenic processes such as cancer progression and metastasis of solid tumors including pancreatic cancer^32^. The involvement of super-enhancers has been invoked to account for squamous-like and metastatic pancreatic cancer phenotypes, and in controlling drug sensitivity in cooperation with H3K4 mono-methylation^32^. It is thought that super-enhancers are linked to the key mechanisms regulating cancer driver genes^33^.

H3K27 acetylation is a functionally important hallmark in the transcriptional regulation of super-enhancers^34^ and a wide variety of oncogenic processes in which it plays a central role have been defined in pancreatic cancer. Diaresis and colleagues reported that H3K27Ac acetylation status and the chromatin landscape can mediate high malignancy and that this can be determined by transcription factor profiling of cancer cells^35^. H3K27 acetylation is also related to enhancer activities to promote cancer metastasis in pancreatic cancer^36^.

In the present study, we investigated the significance of histone H3 acetylation by immunohistochemical staining ex vivo and determined that it was aberrantly activated to different degrees in different patients´ pancreatic cancer tissues (Fig. 6A and Supplementary Figure S2). We found that intermediate levels of acetylated H3K27 expression were associated with a prognosis worse than seen with either hyperacetylation or hypoacetylation (Fig. 6E).

This apparent paradox might depend on permissive epigenetic plasticity, i.e., permissive euchromatin status could pressure cancer cells towards oncogenic gene alterations to acquire phenotypic changes. The acquired potential to promote a malignant phenotype could be mediated by a cell state transition such as the epithelial-mesenchymal transition, and be associated with permissive and plastic chromatin states^37^. Pronounced plasticity is known to be associated with drug resistance by controlling phenotypic states^6,38^. In contrast, a restricted heterochromatin status results in a fixed epigenetic condition insufficient to permit cancer cells to drive extensive gene alterations and oncogenic transformation, as reflected in hyperacetylation and hypoacetylation being associated with a better prognosis in comparison with intermediate histone acetylation status^38^.

Inhibition of HAT activity has already been evaluated as a therapeutic mechanism in several types of cancer such as lung cancer, prostate cancer, and others^13–17^. In terms of the effect of HAT inhibitors on the cell cycle, earlier studies showed that C646 induced cell cycle arrest at G1 in some cancers, such as melanoma, acute myeloid leukemia, and gastric cancer^13,16,17^. In contrast, in our study, C646 treatment reduced the proportion of pancreatic cancer cells in G1 and increased arrest at G2/M, indicating blockade of cell cycle regulator proteins, as shown in Figs. 2A and B and 3A–C. Differences in G1 or G2/M cell cycle arrest on HAT inhibition might therefore be tumor type-specific.

It has been reported that curcumin exerts anti-cancer effects in different histotypes including pancreatic cancer, and that it downregulates histone H3 acetylation^19,39,40^. Curcumin induces G2/M cell cycle arrest in many types of cancer such as breast, colon, and other cancers^41,42^. Curcumin also induced G2/M arrest in pancreatic cancer cell line, BxPC3 cells and led apoptosis^6,43^. In lung cancer, C646 treatment sensitized cancer cells to irradiation by blockade of CHK1 phosphorylation, which is responsible for G2/M entry, and induced mitotic catastrophe on irradiation^15^. We have shown evidence that these HAT inhibitors, such as C646, Curcumin, and Anacardic acid regulate the cell cycle in pancreatic cancer through G2/M cell cycle arrest (Fig. 2A, E).

During the G2/M transition, CDK1 kinase activation is essential for entry into mitosis. This is initiated by binding of cyclin B1 to CDK1, resulting in the formation of M-phase promoting factor^21^. In our study, this multi-step machinery necessary for mitotic entry was blocked because cell cycle regulatory molecules related to G2/M transition, such as cyclin B1 and CDK1 were downregulated at the transcriptional level, and thus did not promote proteasome degradation (data not shown). Owing to the blockade of formation of M-phase promoting factor, the entry of cancer cells into the M phase was prevented by C646 treatment or p300/CBP gene silencing, resulting in G2 cell cycle arrest in pancreatic cancer cells.

In addition, we identified differences in the reactivity of histone acetylation when treated with C646 or CBP/p300 siRNA treatment. C646 served as a HAT inhibitor that globally suppresses H3K9, K18 and K27 acetylation. On the other hand, CBP and p300 downregulation by siRNA treatment suppressed H3K18 and H3K27 acetylation, but not H3K9 acetylation. As reported by Jin et al.^24^, H3K9 acetylation mediates PCAF, while H3K18 and H3K27 acetylation are highly associated with CBP and p300. C646 caused more potent suppression of HAT activity than CBP/p300 dual knockdown, which may account for the difference in CDK1 reduction (Figs. 3A, 4C).

In summary, in the current study, we documented the clinical significance of histone H3 acetylation by immunohistochemical staining. We analyzed the molecular mechanism of action using the HAT inhibitor C646 and showed that HAT inhibition by this agent or CBP/p300 gene silencing effectively blocked G2/M cell cycle transition through the inhibition of the regulatory molecules cyclin B1 and CDK1. Our data indicate that the HAT inhibitor C646 could represent a novel therapeutic agent for pancreatic cancer.

## Methods

### Materials

Anti-p300 (dilution 1:100, N-15; sc-584), anti-CBP (dilution 1:100, A-22; sc-369), anti-PCAF (dilution 1:100, E-8; sc-13124), anti-cyclin B1 (dilution 1:100, GNS1; sc-245), and anti-CDK1 (dilution 1:200, 17; sc-54) antibodies were obtained from Santa Cruz Biochemistry (Santa Cruz, CA). Anti-caspase 3 (dilution 1:1000, 8G10; #9662), anti-cleaved caspase 3 (dilution 1:500, 5A1E; #9664), anti-cleaved PARP (dilution 1:1000, D64E10; #5625), anti-phospho-histone H3 (Ser10) (dilution 1:1000, D2C8; #3377), anti-acetyl histone H3 (Lys9) (dilution 1:1000, C5B11; #9649), anti-acetyl histone H3 (Lys18) (dilution 1:1000, D8Z5H; #13998), anti-acetyl histone H3 (Lys27) (dilution 1:1000, D5E4; #8173), and anti-histone H3 (dilution 1:2000, D1H2; #4499) antibodies were from Cell Signaling Technology (Danvers, MA). Anti-β-actin antibody (dilution 1:2000, AC-15; A5441) was from Sigma-Aldrich (St. Louis, MO). The HAT inhibitor C646 was obtained from Sigma-Aldrich (SML0002) and Selleck Chemicals (S7152) (Houston, TX).

### Cell cultures

The human pancreatic cancer cell lines Hs766T, MIAPaCa2, Panc1, and PSN1 were obtained from the American Type Culture Collection (ATCC, Manassas, VA). The MIAPaCa2, Panc1, and PSN1 lines were obtained in August 2016 and Hs766T in July 2019. All cancer cell lines were authenticated by STR (short tandem repeat) analysis for DNA profiling and all experiments were performed with mycoplasma-free cells. Cancer cells were maintained in high-glucose DMEM medium containing 10% FBS and 1% penicillin/streptomycin in a humidified 5% CO_2_ chamber at 37 °C.

### Human tissues

A total of 102 patients who underwent curative operation for pancreatic cancer at Tokyo Medical and Dental University Hospital between 2007 and 2016. With approval of the ethics committees of the Faculty of Medicine in Tokyo Medical and Dental University (permission No. M2000-1080, G2017-018), written informed consent was obtained from all patients. Patients were anonymously coded in accordance with ethical guidelines, as instructed by the Declaration of Helsinki.

Heat-induced epitope retrieval was performed with citrate buffer (pH 6.0). Anti-acetyl histone H3 (Lys9 and Lys27) antibody was diluted 1/1000 with SignalStain Antibody Diluent (#8112; Cell Signaling Technology) and incubated for 1 h at room temperature. Antigen–antibody reactions were visualized with SignalStain Boost IHC Detection Reagents (HRP, Rabbit #8114; Cell Signaling Technology).

### Western blotting

Western blotting was performed as previously described^44^. Protein bands were visualized, and their intensities quantified using the Odyssey Fc Imaging System (LI-COR Biosciences, Lincoln, NE). β-actin and histone H3 served as loading control markers for normalization of each lane. All exposures for densitometry were within the linear range. Western blotting was repeated at least 3 times with similar results and representative blots are presented. Figures for western blotting are cropped and displayed according to the appropriate molecular weight. Full-length or cropped blots are presented in Supplementary Figure S4.

### Gene silencing by small interfering RNA

Loss-of-function analysis was performed using siRNA targeting p300 (ON-TARGETplus SMARTpool #L-003486-00-0005, Dharmacon, Lafayette, CO) and CBP (ON-TARGETplus SMARTpool #L-003477-00-0005, Dharmacon). ON-TARGETplus Non-Targeting pool siRNA (#D-001810-10, Dharmacon) served as a negative control. Each siRNA (20 nmol/L) was transfected into pancreatic cancer cells using Lipofectamine RNAiMAX (Invitrogen) according to the manufacturer’s instructions. Knockdown of each target gene was confirmed by Western blotting.

### Quantitative real-time RT-PCR

Extracted RNA was reverse transcribed into first-strand cDNA using SuperScript VILO cDNA Synthesis kits (Invitrogen, Carlsbad, CA). Expression of mRNA was determined using TaqMan Gene Expression Assays (Applied Biosystems, Foster City, CA). The TaqMan assays used in this study were CCNB1; Hs01030099_m1, CDK1; Hs00938777_m1, EP300; Hs00914223_m1, CREBBP; Hs00231733_m1, and KAT2B; Hs00187332_m1. Gene expression values are presented as ratios between genes of interest and an internal reference gene (Hs99999901_s1 for eukaryotic 18S), and subsequently normalized against the value for the control (relative expression level). Each assay was performed in duplicate for each sample.

### Cell viability and BrdU incorporation assays

Cell numbers were evaluated by an assay based on a colorimetric water-soluble tetrazolium salt, WST-8 (Cell Counting Kit-8; Dojindo Molecular Technologies, Gaithersburg, MD), as previously described^45^. A second cell proliferation assay used BrdU Cell Proliferation Assay Kits (Cell Signaling Technology) according to the manufacturer's instructions. The absorbance of each well was measured at 450 nm using a Quant Microplate Spectrophotometer (BioTek Instruments, Winooski, VT).

### Clonogenic assay

Long-term cell survival was evaluated by clonogenic assays as previously described^23^. Cells at 1.0 × 10^3^ per well were seeded into 6-well plates in triplicate. After overnight incubation, adherent cells were treated with C646 for 9 h, after which culture medium was subsequently changed to fresh medium without C646. Cells were incubated for at least 1 week, and colonies were then stained with 0.3% crystal violet solution. A cluster of ≥ 50 stained cells was counted as a colony.

### Apoptosis assay

Apoptotic cells were quantified by flow cytometry (LSRII; BD Bioscience, San Diego CA) following annexin V-FITC and propidium iodide (PI) staining using Annexin V-FITC Apoptosis Detection Kits (Sigma).

### Cell cycle analysis

Cells were fixed in 70% ethanol at -20 °C overnight. To determine the DNA content, cells were stained with 50 µg/mL PI (Sigma Aldrich) with Triton X-100 and RNAase and then analysed by flow cytometry (LSRII). At least 10,000 events for each sample were acquired and analyzed with FlowJo software (Tree Star, Ashland, OR).

### Chromatin immunoprecipitation

Chromatin immunoprecipitation (ChIP) assays were performed using a ChIP-IT Express Kit (Active Motif, Carlsbad, CA) according to manufacturer’s protocol as previously described^46^. DNA samples were prepared from non-treated and C646-treated PSN1 cells. C646 treatment was performed at 40 µM for 48 h. Cells were fixed 1% formaldehyde/PBS for 10 min. The DNA was sonicated to 500–1000 bp in all experiments. Immunoprecipitated DNA enrichment was normalized to the input. The antibodies were anti-H3K9ac (dilution 1:50, #9649; Cell Signaling Technology), anti-H3K27ac (dilution 1:50, #8173; Cell Signaling Technology) and anti-histoneH3 (1 µg, #07-690; Millipore). Normal rabbit IgG (#2729; Cell Signaling Technology) was used as a negative control for each assay. The primer sets for quantitative PCR are listed in Supporting Information Table S2.

### Mouse xenograft model

Five-week-old nude male mice were purchased from Charles River Laboratories. Mice were inoculated subcutaneously at their flank (MIAPaCa2 cells, 7.5 × 10^6^ cells per mouse). After 10 days, treatment commenced when tumors reached a diameter of approximately 5 mm. Mice bearing tumors were treated with C646 (10 mg/kg) or control vehicle (DMSO) by daily i.p. injection for 2 weeks, or went without treatment (non-treated controls). The size of the growing tumors was monitored every 3 days. Tumor volumes were calculated using the following formula: volume = ½ a × b^2^, where a and b represent the larger and smaller tumor diameters, respectively. All mouse procedures were approved by the Institutional Animal Care and Use Committee of Tokyo Medical and Dental University (permission No. A2019-263C2) and conducted under the relevant guidelines and regulations established by it. For all mouse experiments, authors complied with the ARRIVE guidelines.

### Statistical analysis

Clinicopathological factors were compared by Mann–Whitney U test and Chi-squared test. Multivariate analysis used a logistic regression model. The cumulative survival rate was estimated using the Kaplan–Meier method, and significance was determined by log-rank test. Statistical analyses were performed using SPSS for Windows, version 25.0 (SPSS Inc., Chicago, IL). Figures were created and statistical analyses performed with GraphPad Prism 7 software (GraphPad Software Inc., San Diego, CA). Unless otherwise specified, independent experiments were conducted in triplicate and the values presented represent their means, compared using Student’s t-test for single comparisons or ANOVA with post hoc Dunnett’s test for multiple comparisons as appropriate. *P* values < 0.05 were considered significant.

## Supplementary Information

**Supplementary Information**.
