# Supplementary material for: C646 inhibits G2/M cell cycle-related proteins and potentiates anti-tumor effects in pancreatic cancer
Source: Sci Rep. 2021 May 12;11:10078. doi: 10.1038/s41598-021-89530-8 (PMC8115044; doi:10.1038/s41598-021-89530-8)
Supplement: Supplementary file 1 — Supplementary Information. [file 41598_2021_89530_MOESM1_ESM.pdf]

Title:

C646 inhibits G2/M cell cycle-related proteins and potentiates anti-tumor effects in pancreatic cancer

Authors:

*Hiroaki Ono[1,2], Tomotaka Kato[1], Yoshiki Murase[1], Yutaro Nakamura[1], Yoshiya Ishikawa[1], Shuichi Watanabe[1], Keiichi Akahoshi[1], Toshiro Ogura[1], Kosuke Ogawa[1], Daisuke Ban[1], Atsushi Kudo[1], Yoshimitsu Akiyama[3], Shinji Tanaka[3], Hiromichi Ito[2] and Minoru Tanabe[1]

[1] Department of Hepatobiliary and Pancreatic Surgery, Graduate School of Medicine, Tokyo Medical and Dental University

Tokyo, JAPAN

[2] Department of Surgery

Michigan State University, College of Human Medicine

Lansing, MI, USA

[3] Division of Molecular Oncology, Graduate School of Medicine, Tokyo Medical and Dental University

Tokyo, JAPAN

All correspondence: **to**:

*Hiroaki Ono, MD, PhD

Department of Hepatobiliary and Pancreatic Surgery, Graduate School of Medicine, Tokyo Medical and Dental University

Address: 1-5-45 Yushima, Bunkyo-ku, Tokyo 113-8519, Japan

Telephone number: +81-3-5803-5928; Fax number: +81-3-5803-0263

E-mail address: ono.msrg@tmd.ac.jp

Key words:

Cell cycle, C646, histone acetyltransferase, pancreatic cancer, H3K27Ac

Word count: 3937 words

Running title:

HAT inhibition to treat pancreatic cancer

Supplementary Figure S1

A

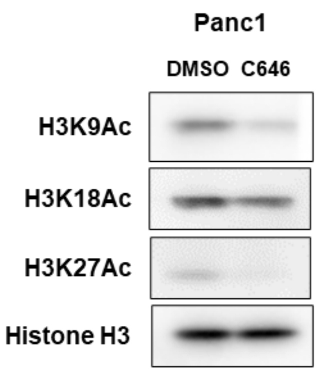

B

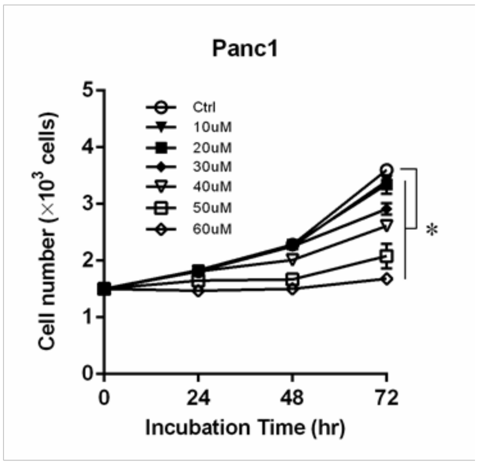

C

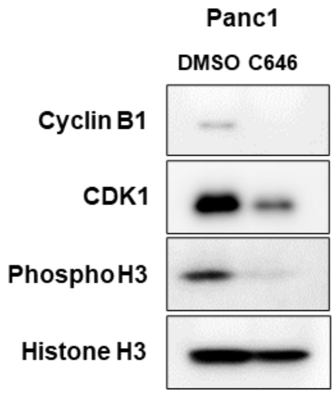

Supplementary Figure S2

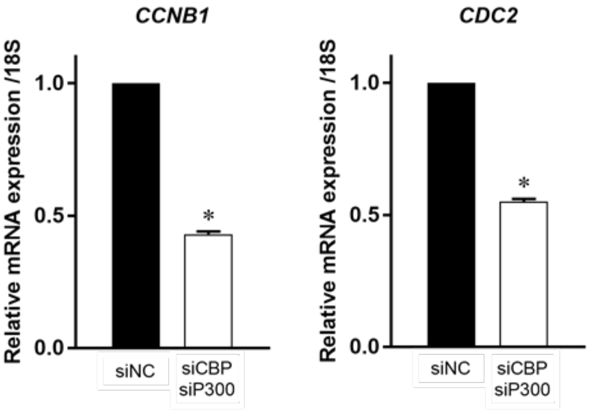

Supplementary Figure S3

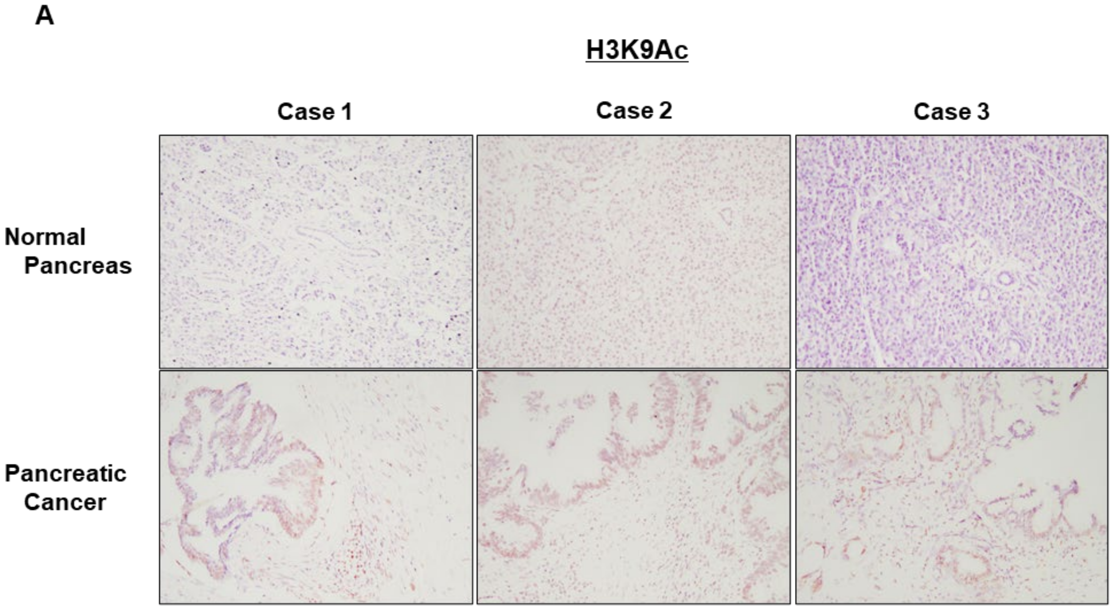

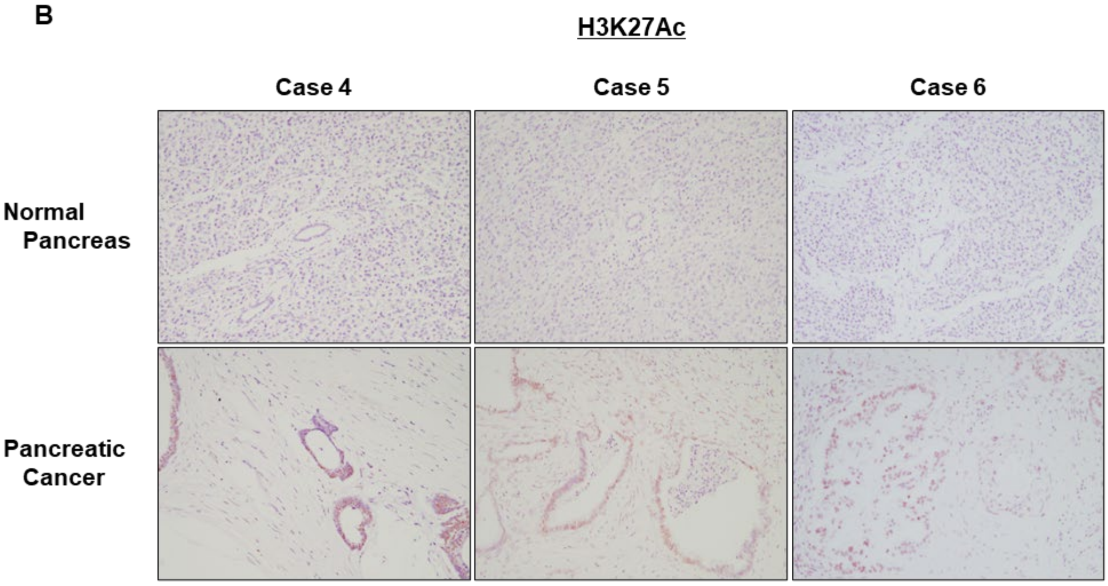

Supplementary Table S1

Supplementary Table 1. Cox proportional hazard analysis for the prognostic factors of overall survival

**Characteristics**	**Univariate analysis**		**Multivariate analysis**	
	HR (95% CI)	*p* - value	HR (95% CI)	*p* - value
Age (>75 vs <75)	1.23 (0.74 – 2.05)	NS	-	-
Sex (Male vs Female)	1.07 (0.66 - 1.73)	NS	-	-
CEA, ng/ml (≥5 vs <5)	1.55 (0.95 - 2.52)	NS	-	-
CA19-9, U/ml (≥100 vs <100)	1.56 (0.98 – 2.47)	NS	-	-
Tumor location (Head vs Body/Tail)	1.39 (0.84 – 2.30)	NS	-	-
Lymphatic Invasion (Positive vs Negative)	2.72 (1.58 – 4.70)	< 0.001	1.61 (0.86 – 2.99)	0.134
Venous Invasion (Positive vs Negative)	2.72 (1.58 – 4.70)	< 0.001	2.28 (1.12 – 4.62)	0.023
Residual Tumor (Positive vs Negative)	1.59 (0.96 – 2.63)	NS	-	-
T status (AJCC7) (≥ T3 vs <T3)	1.56 (0.96 – 2.53)	NS	-	-
N status (AJCC7) (≥ N1 vs N0)	3.35 (1.80 – 6.24)	<0.001	1.72 (0.82 – 3.59)	0.149
H3K27Ac expression (Intermediate vs Others)	1.67 (1.05 – 2.61)	0.03	1.57 (0.97 – 2.53)	0.066

CI: confidence interval, CEA: carcinoembryonic antigen, CA19-9: carbohydrate antigen 19-9,
AJCC7: American Joint Commission on Cancer 7th edition

Supplementary Table S2.

Primer sequences for qChIP-PCR.

Gene	Sense	Antisense
CCNB1	5'-ATCGCCCTGGAAACGCATTCTCT-3'	5'-AGAAGCAGAACACCGGAGGC-3'
CDC2	5'-CCCTTTAGCGCGGTGAGTTTG-3'	5'-CAGCTACAACAACCGCGTCG-3'

Supplementary Figure legends

Supplementary Figure S1

A. Effects of C646 treatment on histone H3 acetylation and expression profiles of molecules associated with the G2/M transition in Panc1 cells with low expression of histone H3 acetylation. Acetylated H3K9 and H3K27were downregulated by the treatment. Cancer cells were treated with 40 μM C646 for 72 hours.

B. Cell viability following C646 treatment of Panc1 cells by WST-8 assay. Cells were treated with titrated doses of C646 (10-60 μM). Cell viability assays were performed every 24 hours up to 72 hours. C646 treatment was for 72 hours. Each data point was evaluated as relative % ratio normalized to vehicle control. Each experiment was performed in duplicate. Error bars represent mean ± SD. *p<0.05 by one-way ANOVA with post hoc Dunnett's test.

C. Protein expression of cyclin B1, CDK1, and phosphorylated histone H3 (Ser10) was also inhibited by the treatment.

Supplementary Figure S2

Expression of mRNA for molecules associated with the G2/M transition following treatment with CBP- and p300- specific siRNAs. Cyclin B1 and CDK1 mRNA expression

was inhibited after CBP/p300 siRNA treatment for 72 hours in MIAPaCa2 cells. Error bars represent mean ± SD. *$p<0.05$ vs controls treated with negative control siRNA.

Supplementary Figure S3

A, B. Representative immunohistochemical staining of pancreatic cancer patients with positive H3K9Ac**(A)** and H3K27Ac**(B)** expression by comparing normal tissues to pancreatic cancer. Upper panel: adjacent normal pancreatic tissue. Lower panel: pancreatic cancer.

Supplementary Figure S4

Figures for western blotting are cropped and displayed according to the appropriate molecular weight.

Supplementary Figure S4

Figure1A

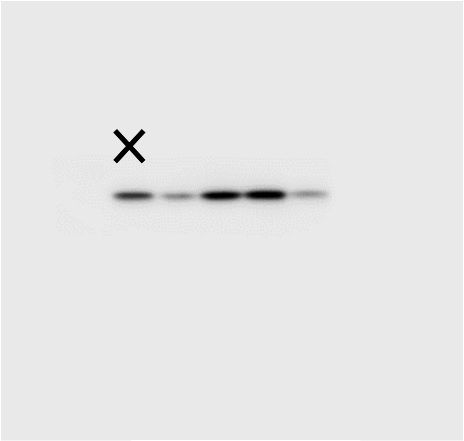

H3K9Ac

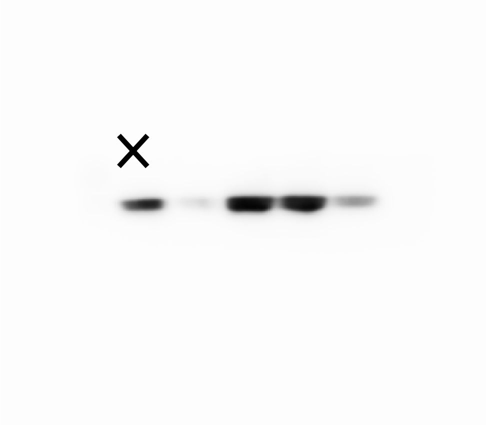

H3K18Ac

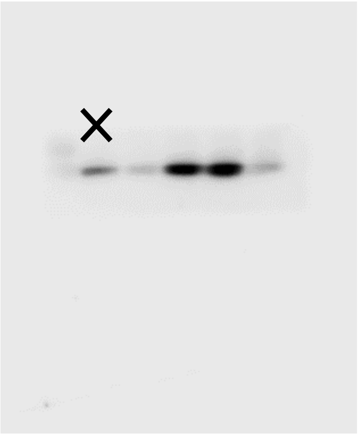

H3K27Ac

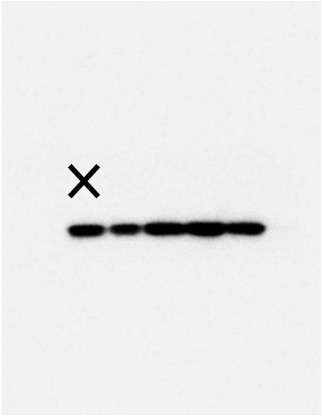

Histone H3

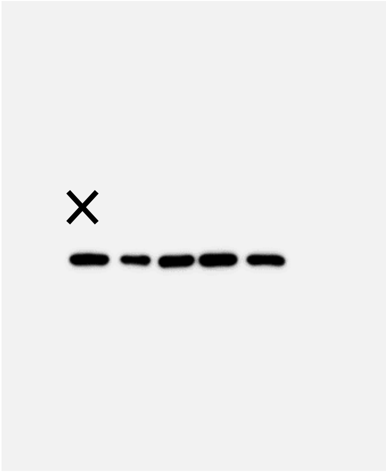

β-actin

Figure 1

A. Endogenous H3K9Ac, H3K18Ac, and H3K27Ac expression in human pancreatic cancer cell lines. Histone H3 acetylation levels were quantified in four pancreatic cancer cell lines, Hs766T, MIAPaCa2, PSN1, and Panc1, by Western blotting.

Figure1B

PSN1 H3K9Ac

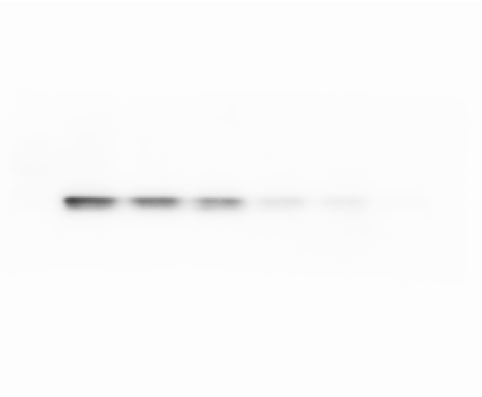

PSN1 H3K18Ac

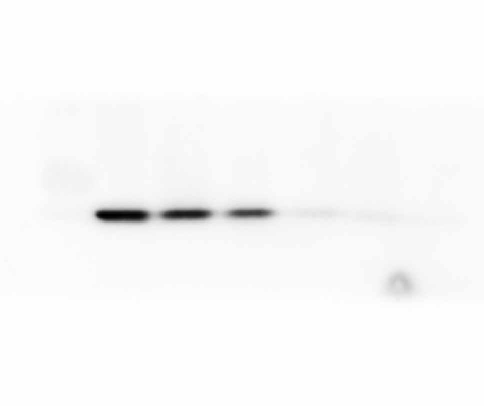

PSN1 H3K27Ac

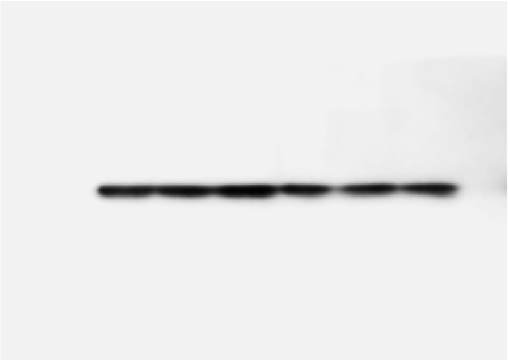

PSN1 HistoneH3

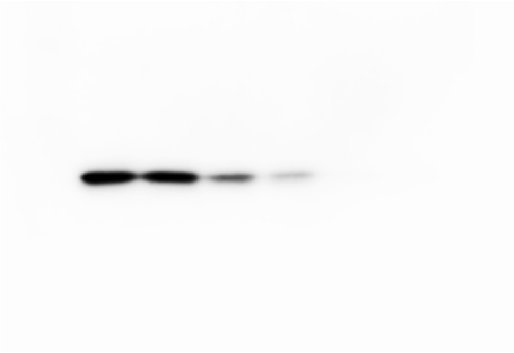

MIAPaCa2 H3K9Ac

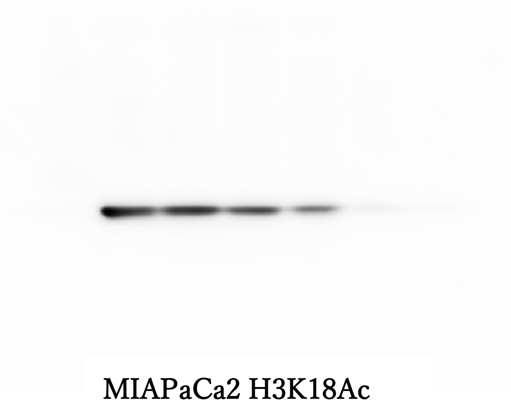

MIAPaCa2 H3K18Ac

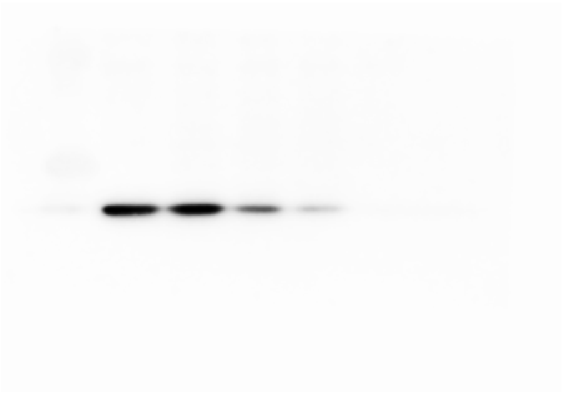

MIAPaCa2 H3K27Ac

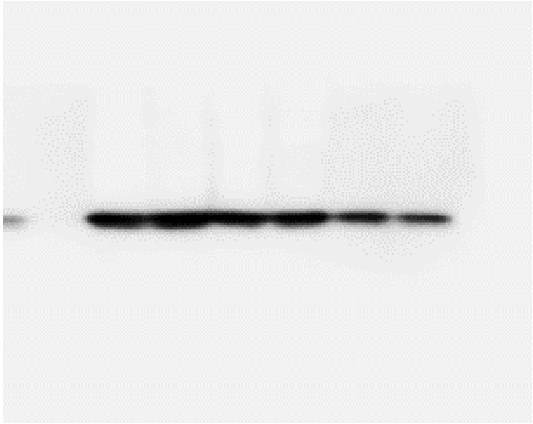

MIAPaCa2 Histone H3

Figure1

B. C646 treatment (10-50 μM) compared with the DMSO vehicle control by Western blotting of PSN1 and MIAPaCa2 cells. Histone H3K9, H3K18, and H3K27 acetylation were downregulated as the C646 concentration increased. Experiments were performed in duplicate.

Figure2D

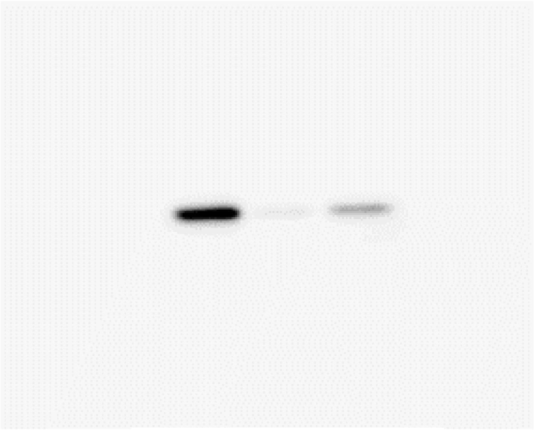

PSN1 H3K9Ac

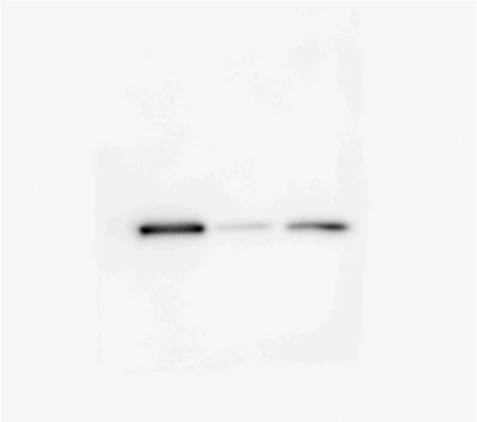

PSN1 H3K18Ac

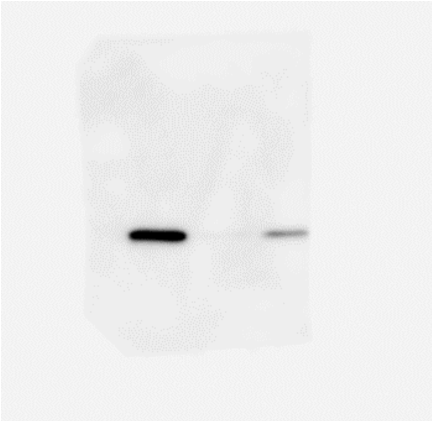

PSN1 H3K27Ac

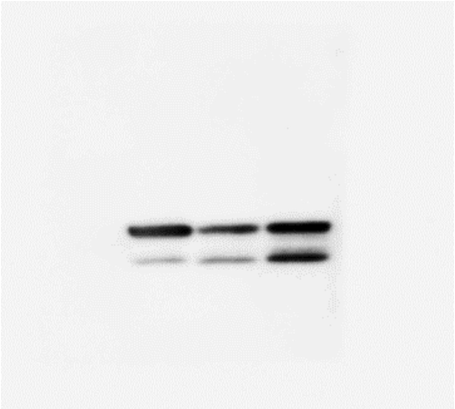

PSN1 Histone H3

Figure2

D. Effects of curcumin and anacardic acid on histone H3 acetylation. Acetylation of H3K9, H3K18, and H3K27 were assessed following 72-hour treatment of curcumin and anacardic acid. Histone H3 acetylation of H3K9, H3K18, and H3K27 were effectively downregulated by curcumin (30 μM) and anacardic acid (100 μM).

Figure3A

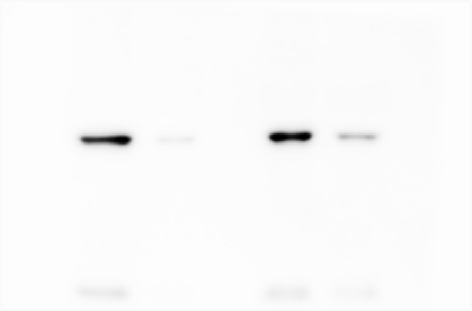

PSN1 MIAPaCa2 CyclinB1

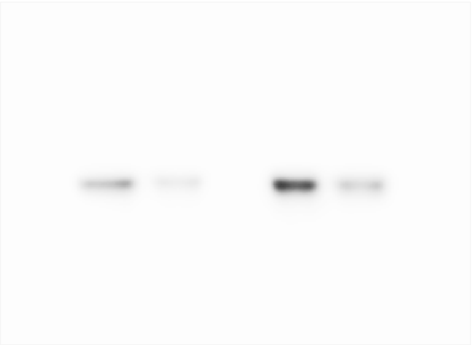

PSN1 MIAPaCa2 CDK1

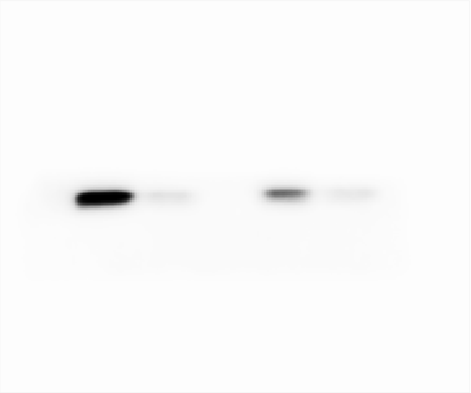

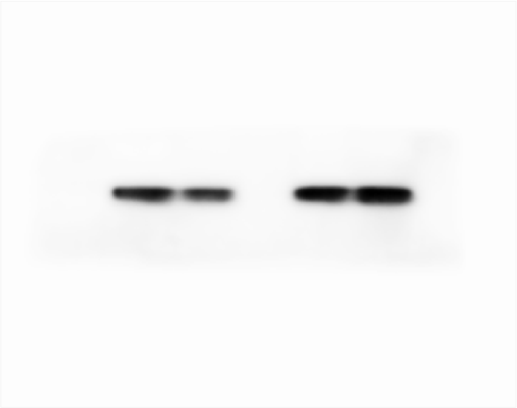

PSN1 MIAPaCa2 phospho H3

PSN1 MIAPaCa2 Histone H3

Figure3A

A, B. Expression profiles of molecules associated with the G2/M transition at the protein level **(A)** and mRNA level **(B)** following C646 treatment. mRNA expression of cyclin B1 and CDK1 was inhibited after 48-hour treatment with 30 μM C646 treatment in PSN1 and MIAPaCa2 cells. Inhibition of protein expression of cyclin B1 and CDK1 after 72-hour C646 treatment was confirmed by Western blotting. Phosphorylated histone H3 (Ser10), recognized as an M phase marker, was also inhibited by C646 treatment.

Figure3C

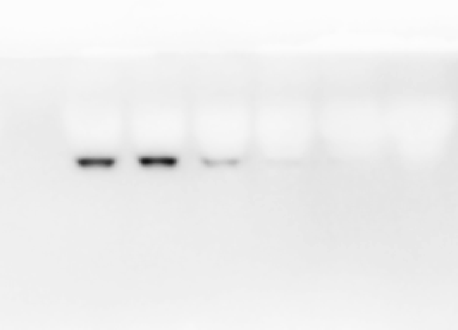

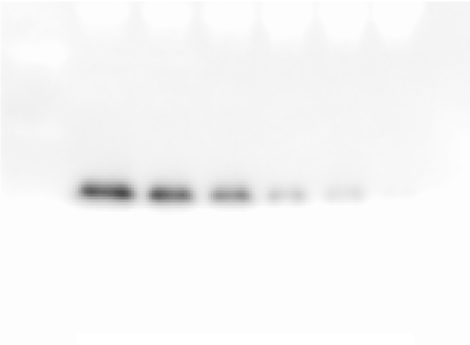

PSN1 CyclinB1

PSN1 CDK1

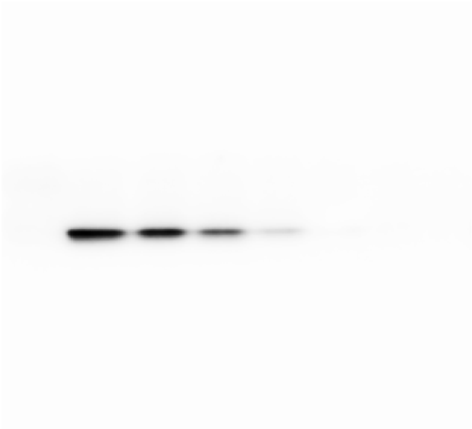

PSN1 PhosphoH3

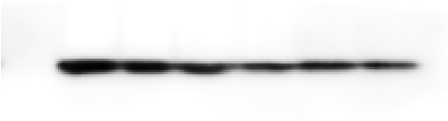

PSN1 HistoneH3

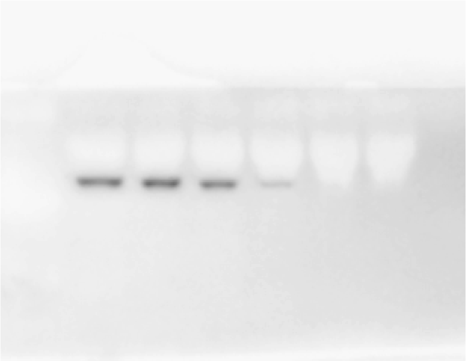

MIAPaCa2 CyclinB1

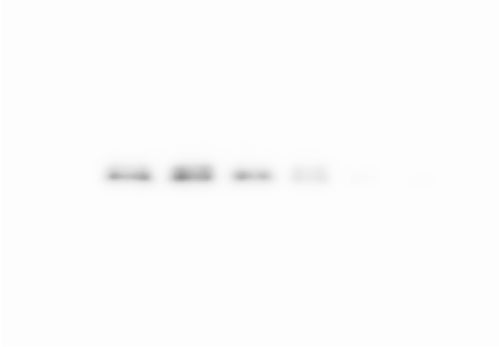

MIAPaCa2 CDK1

MIAPaCa2 PhosphoH3

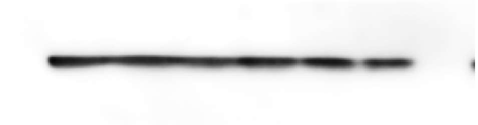

MIAPaCa2 HistoneH3

Figure3C

C. Dose-dependent analysis of G2/M cell cycle-associated molecules following C646 treatment (10-50 μM) by Western blotting in PSN1 and MIAPaCa2 cells.

Figure3D

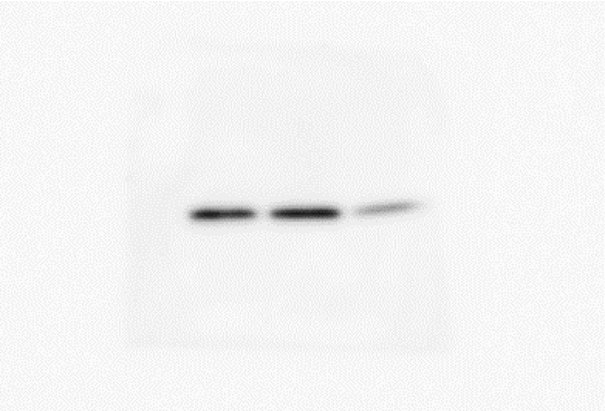

PSN1 H3K9Ac

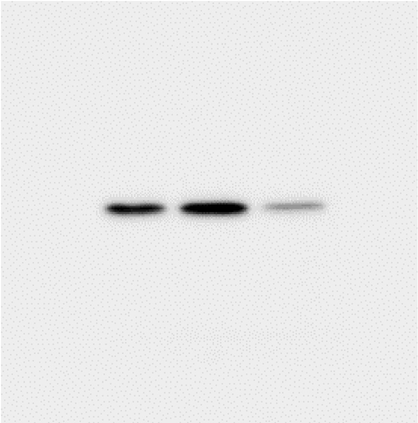

PSN1 H3K18Ac

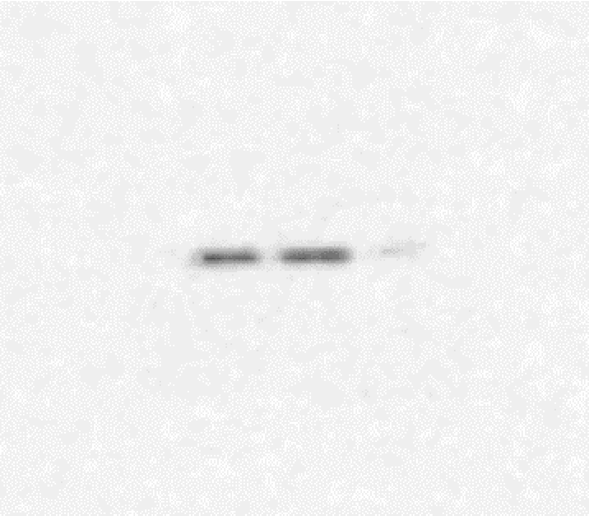

PSN1 H3K27Ac

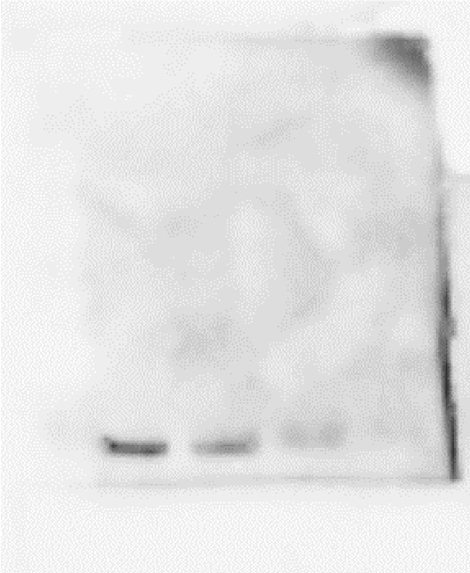

PSN1 cyclin B1

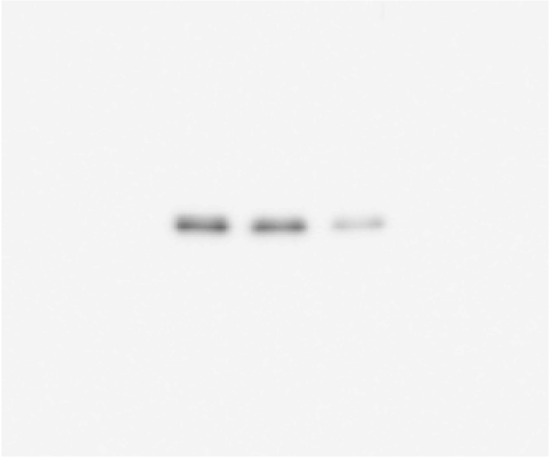

PSN1 CDK1

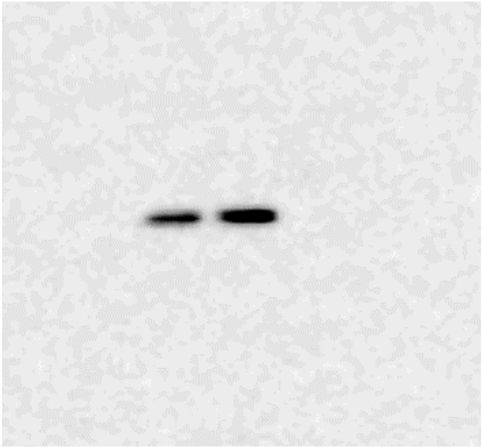

PSN1 phospho H3

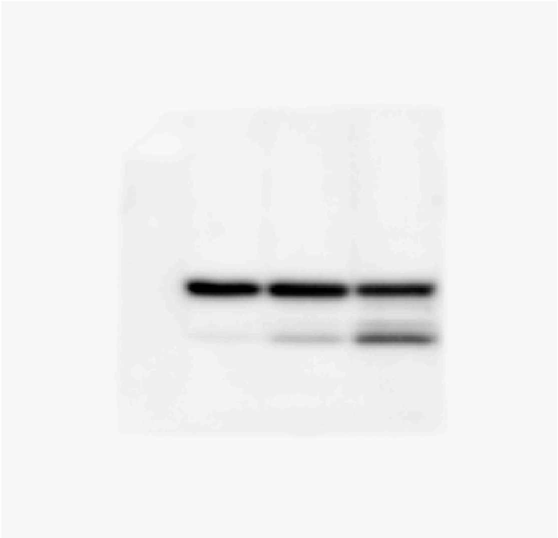

PSN1 Histone H3

Figure3D

D. Time-dependent analysis of histone H3 acetylation and G2/M cell cycle-associated molecules following C646 treatment for 24 and 48 hours in PSN1 cells.

Figure4C

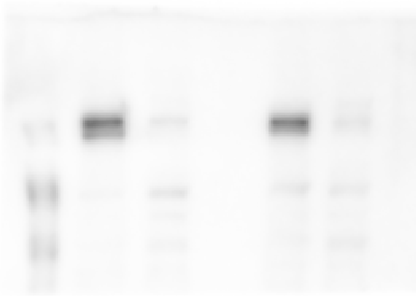

PSN1 MIAPaCa2 CBP

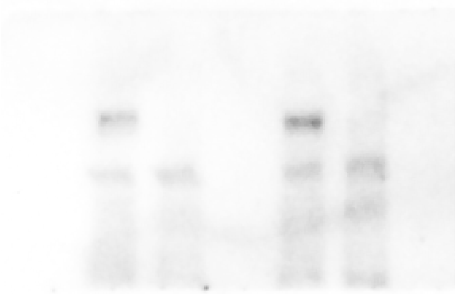

PSN1 MIAPaCa2 p300

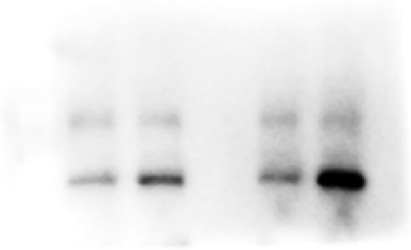

PSN1 MIAPaCa2 PCAF

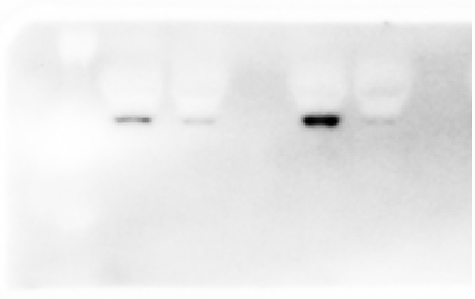

PSN1 MIAPaCa2 cyclin B1

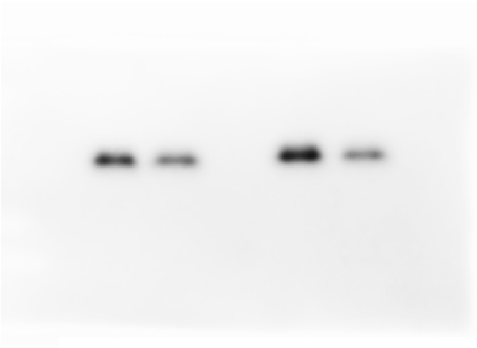

PSN1 MIAPaCa2 CDK1

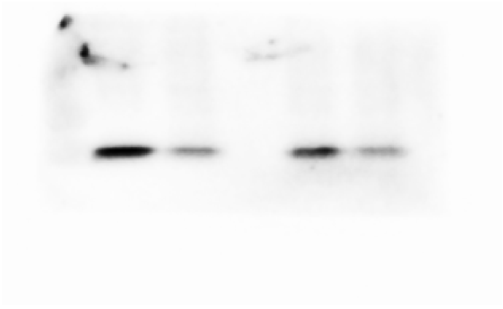

PSN1 MIAPaCa2 phospho H3

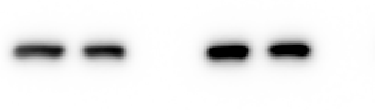

PSN1 MIAPaCa2 β-actin

Figure4C

C. Effects on CBP, p300, and PCAF expression and expression of G2/M cell cycle regulatory molecules after p300 and CBP siRNA treatment. Effective knockdown of CBP and p300 by treatment with CBP- and p300-specific siRNAs was confirmed at the protein level. PCAF expression was markedly increased by p300 and CBP gene silencing. Expression of G2/M cell cycle regulatory molecules were suppressed. Cancer cells were treated with CBP- and p300-specific siRNAs or negative control siRNA for 72 hours.

Figure4D

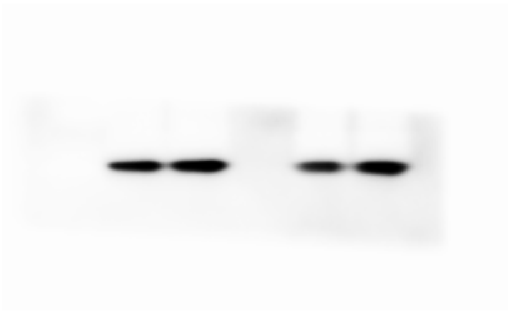

PSN1 MIAPaCa2 H3K9Ac

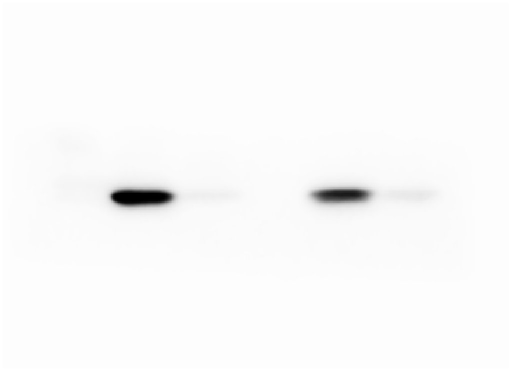

PSN1 MIAPaCa2 H3K18Ac

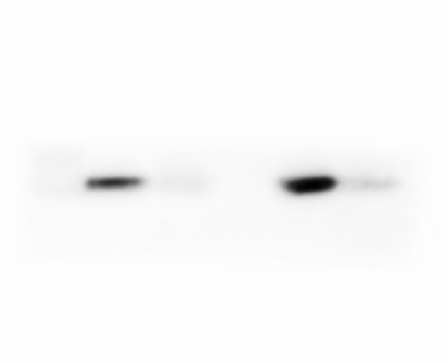

PSN1 MIAPaCa2 H3K27Ac

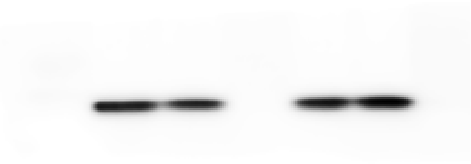

PSN1 MIAPaCa2 HistoneH3

Figure4D

D. Effects of silencing both p300 and CBP genes on histone H3 acetylation. Effects on acetylation of H3K9, H3K18, and H3K27 were assessed. Acetylated H3K18 (H3K18Ac) and H3K27 (H3K27Ac) were downregulated by CBP and p300 gene silencing, while acetylated H3K9 (H3K9Ac) was not affected.

Figure4E

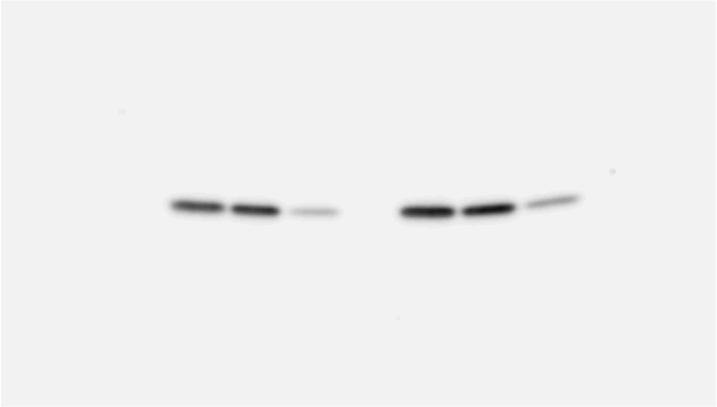

PSN1 MIAPaCa2 H3K9Ac

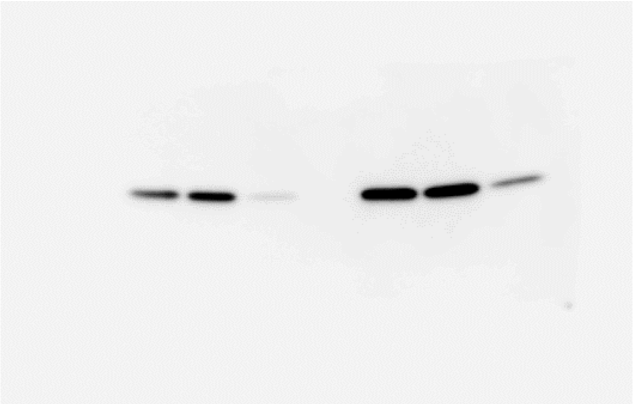

PSN1 MIAPaCa2 H3K18Ac

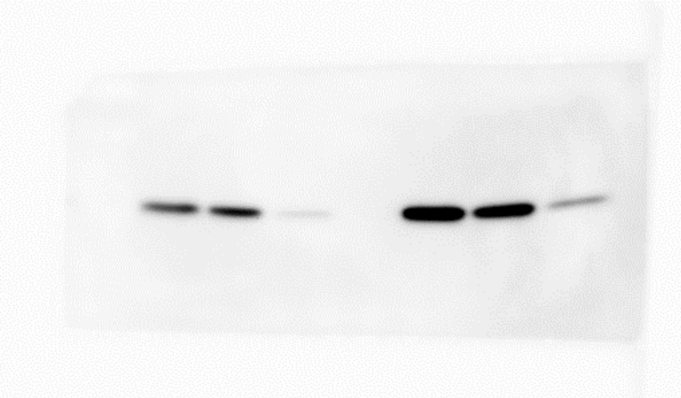

PSN1 MIAPaCa2 H3K27Ac

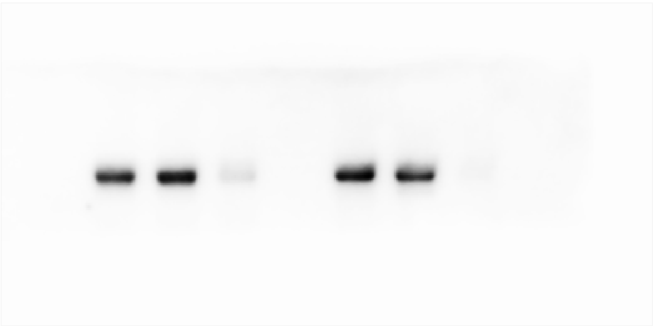

PSN1 MIAPaCa2 CBP

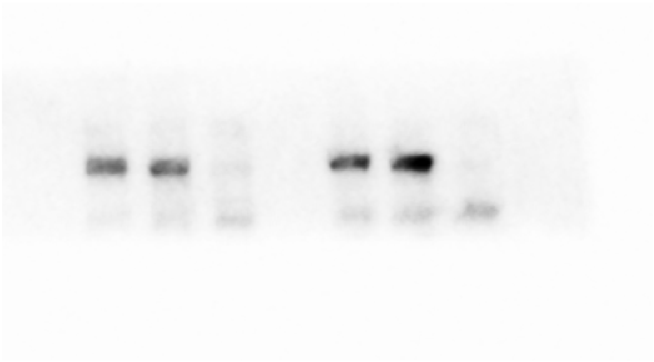

PSN1 MIAPaCa2 p300

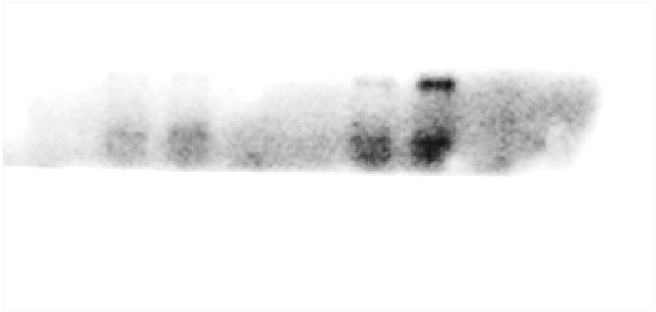

PSN1 MIAPaCa2 PCAF

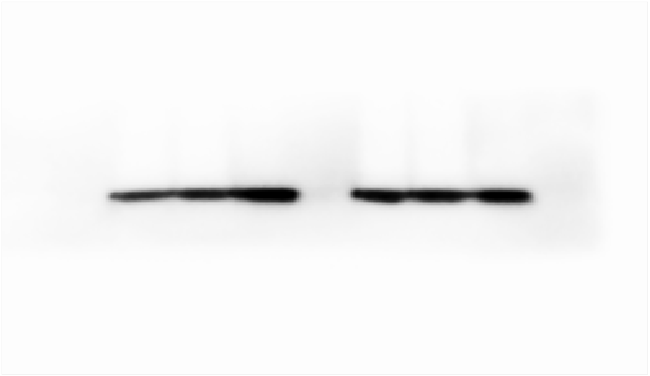

PSN1 MIAPaCa2 Histone H3

Figure4E

E. Effects of C646 treatment on histone H3 acetylation and CBP, p300, and PCAF expression in PSN1 and MIAPaCa2 cells. Cancer cells were treated with C646 for 72 hours at each concentration (20 and 40 μM). At a C646 concentration of 40 μM, which sufficiently inhibited histone acetylation, expression of these HAT molecules was downregulated.

Figure5B

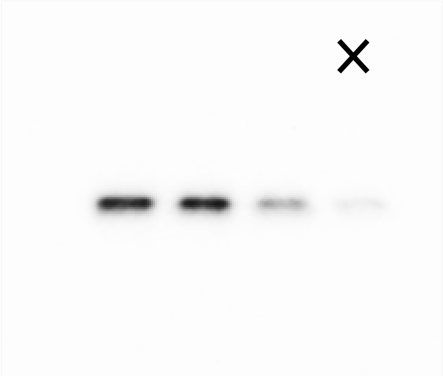

PSN1 Caspase3

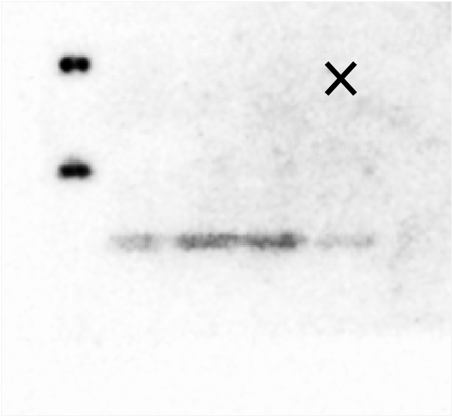

PSN1 Cl Caspase3

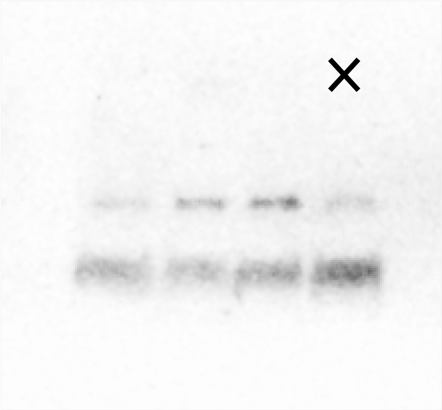

PSN1 Cl PARP

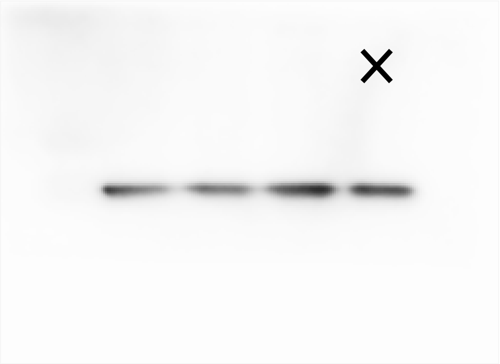

PSN1 Histone H3

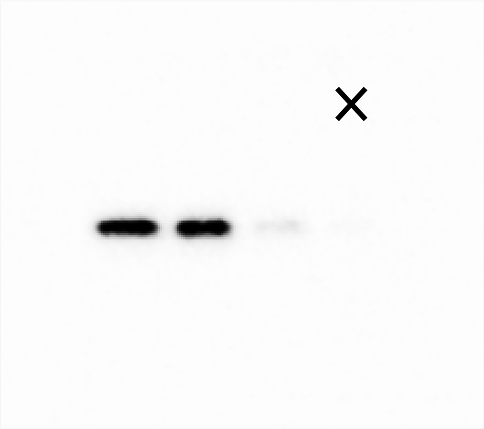

MIAPaCa2 Caspase3

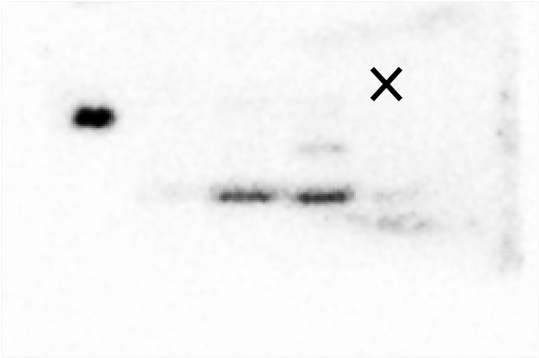

MIAPaCa2 Cl Caspase3

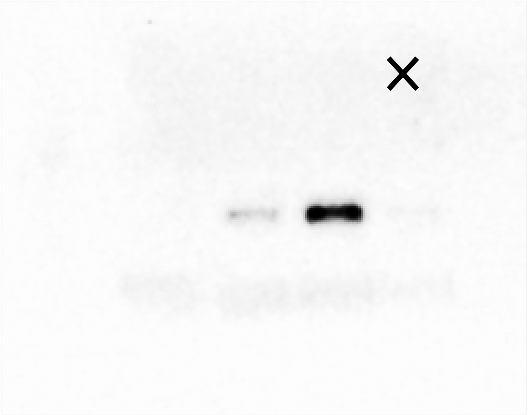

MIAPaCa2 Cl PARP

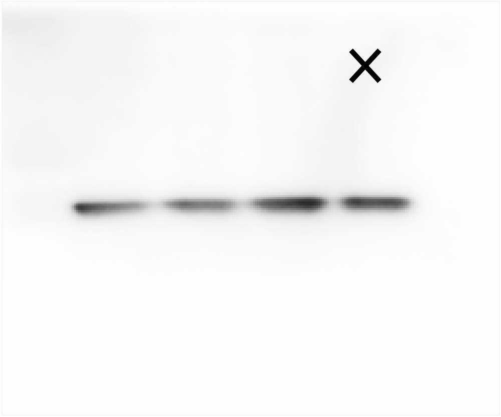

MIAPaCa2 Histone H3

Figure5B

B. Cancer cells were treated with C646 at 30 μM for 48 hours. The expression of apoptotic markers was increased by C646 treatment compared with controls.

Supplementary Figure S1A

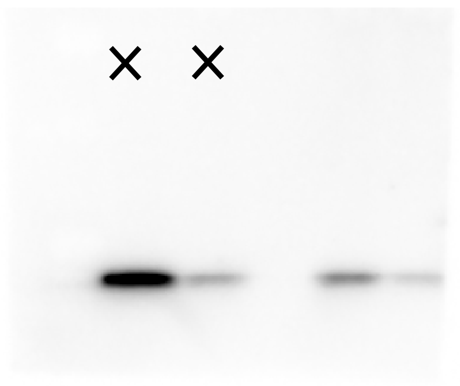

MIAPaCa2 Panc1 H3K9Ac
MIAPaCa2: not used

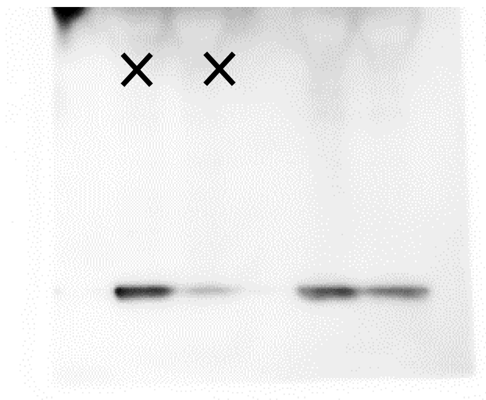

MIAPaCa2 Panc1 H3K18Ac
MIAPaCa2: not used

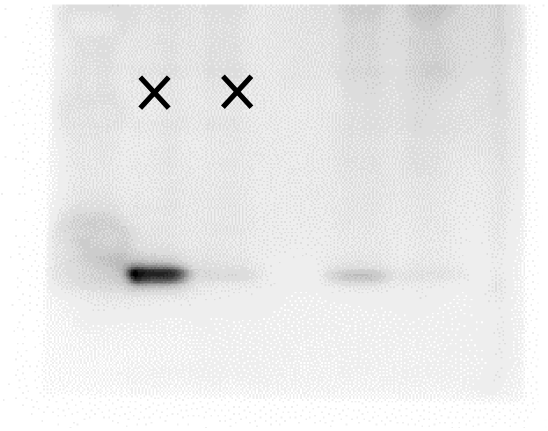

MIAPaCa2 Panc1 H3K27Ac
MIAPaCa2: not used

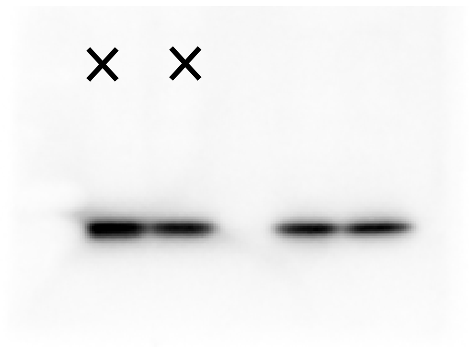

MIAPaCa2 Panc1 Histone H3
MIAPaCa2: not used

Supplementary Figure S1C

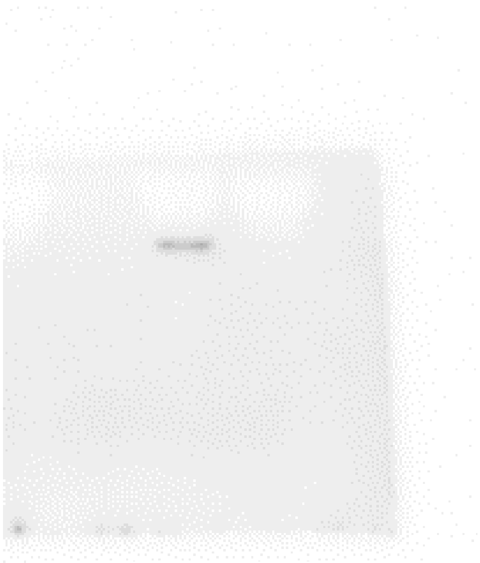

Panc1 cyclin B1

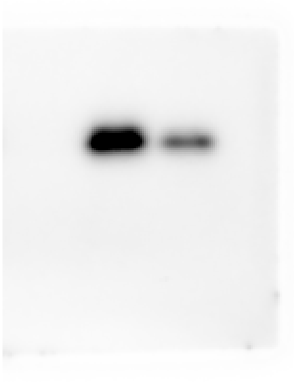

Panc1 cyclin CDK1

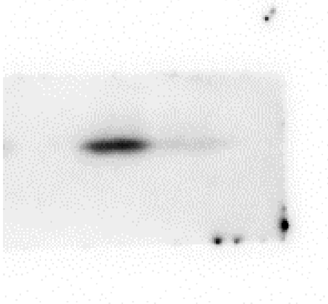

Panc1 phospho H3

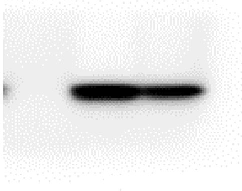

Panc1 Histone H3

Figure1B for densitometry

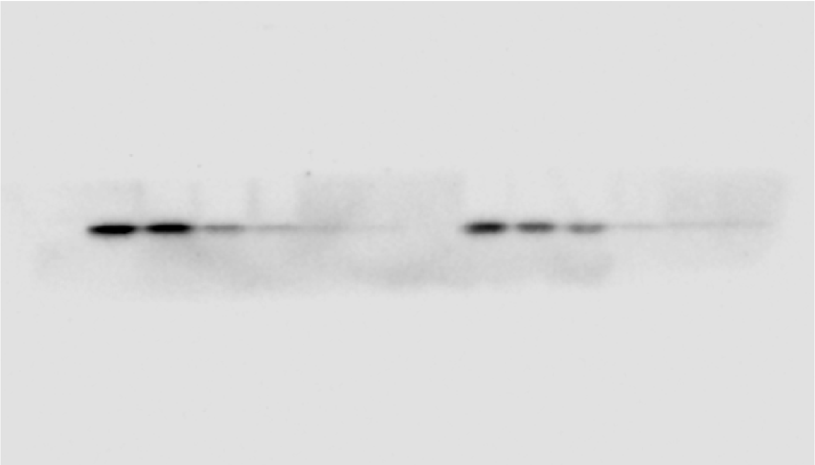

MIAPaCa2 PSN1 H3K9Ac

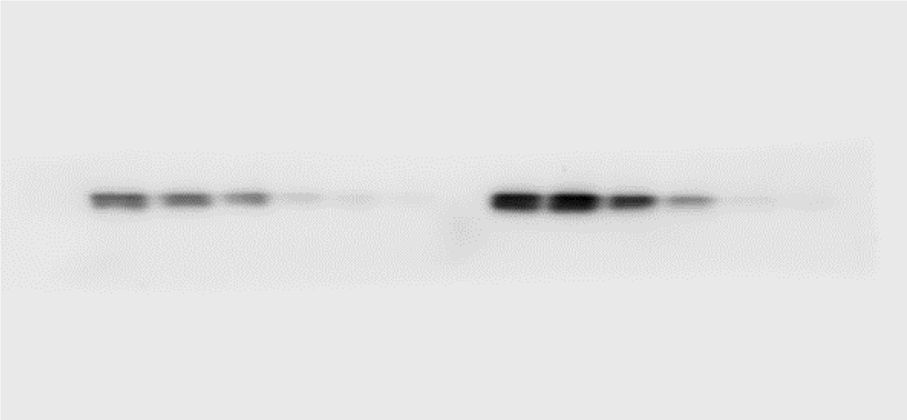

PSN1 MIAPaCa2 H3K18Ac

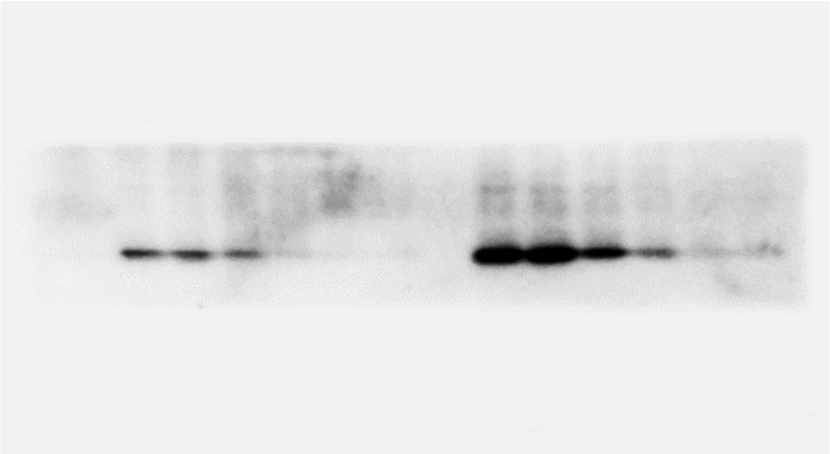

PSN1 MIAPaCa2 H3K27Ac

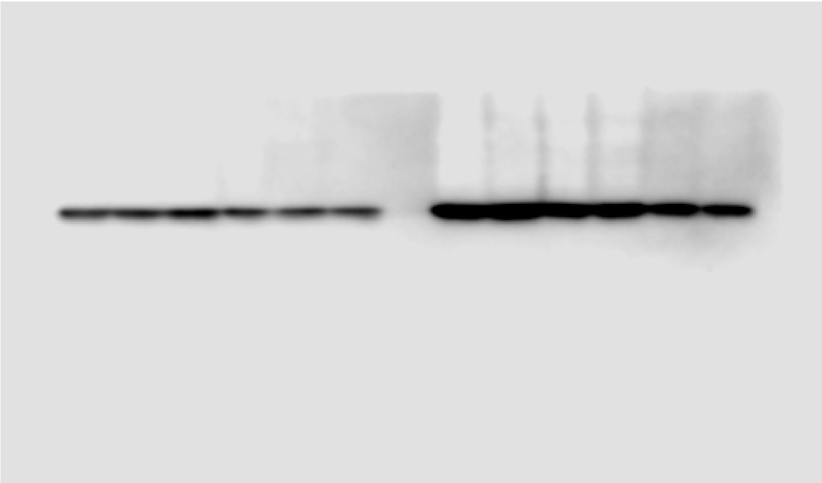

PSN1 MIAPaCa2 Histone H3

Figure4C for reproducilibity

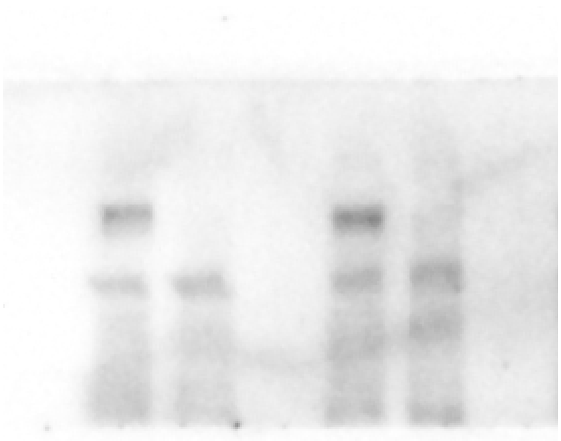

PSN1 MIAPaCa2 siNC, siP300/CBP
p300

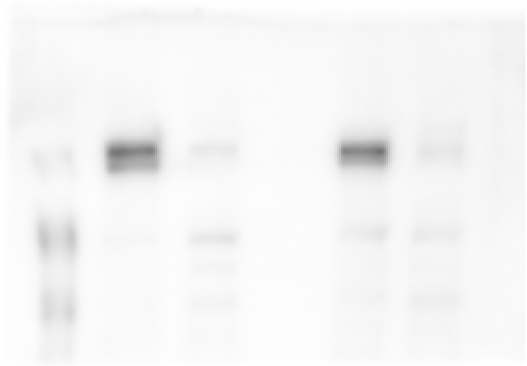

PSN1 MIAPaCa2 siNC, siP300/CBP
CBP

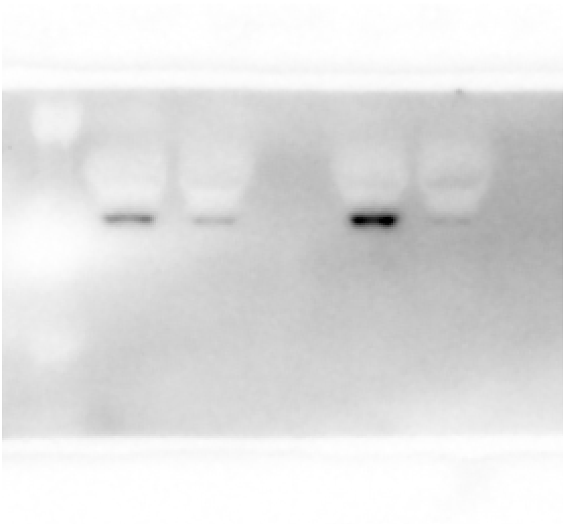

PSN1 MIAPaCa2 siNC, siP300/CBP
cyclin B1

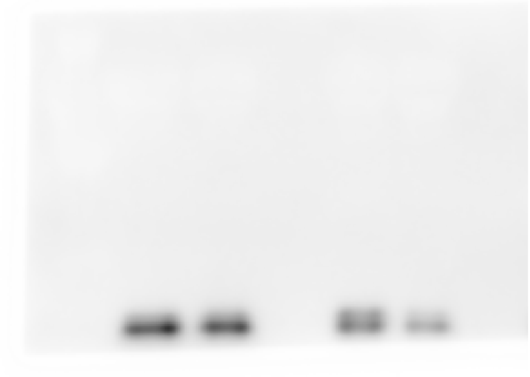

PSN1 MIAPaCa2 siNC, siP300/CBP
CDK1CBP

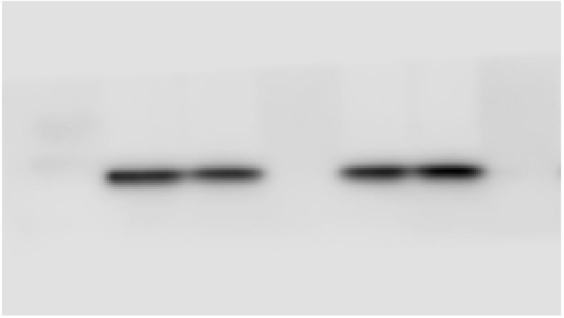

PSN1 MIAPaCa2 siNC, siP300/CBP
Histone H3

Figure4E for reproducilibity

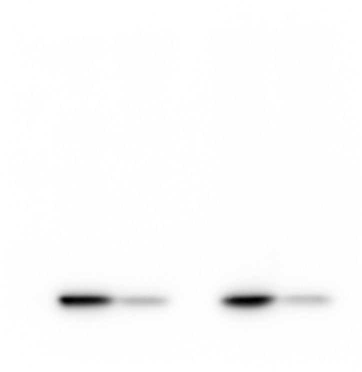

PSN1 MIAPaCa2
DMSO/C646 30uM H3K9Ac

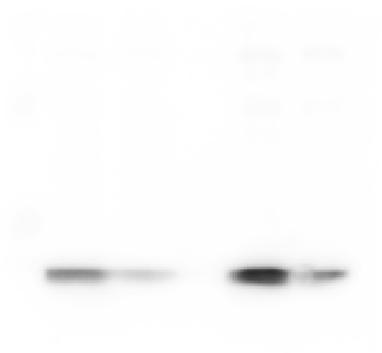

PSN1 MIAPaCa2
DMSO/C646 30uM H3K18Ac

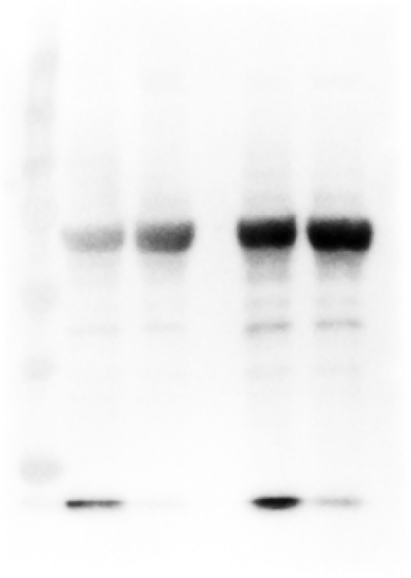

PSN1 MIAPaCa2
DMSO/C646 30uM H3K27Ac

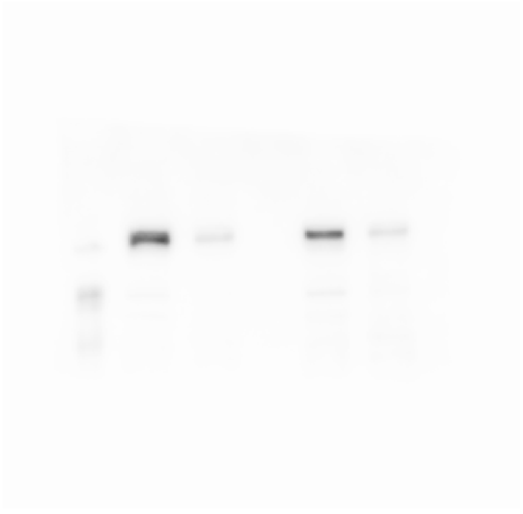

PSN1 MIAPaCa2
DMSO/C646 30uM CBP

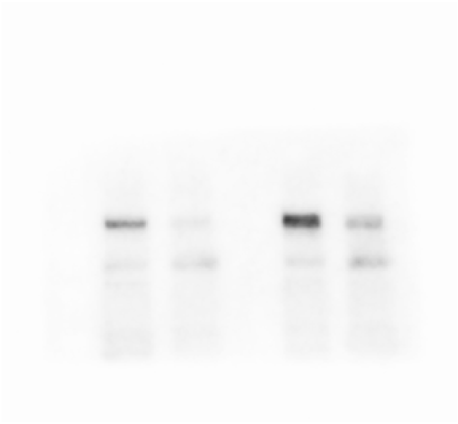

PSN1 MIAPaCa2
DMSO/C646 30uM p300

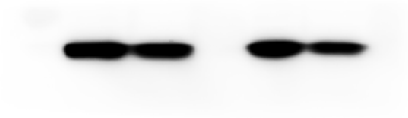

PSN1 MIAPaCa2
DMSO/C646 30uM Histone H3

Figure5B for reproducilibity

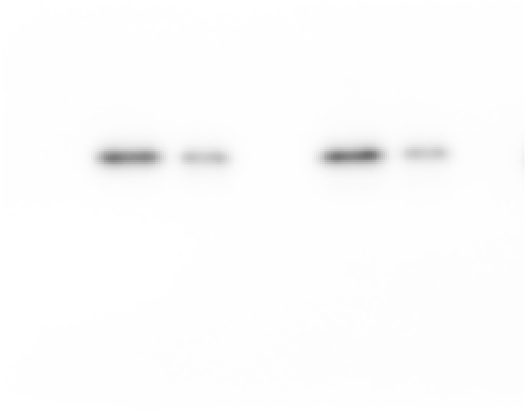

PSN1 MIAPaCa2
DMSO/C646 30uM, 48 hours Caspase3

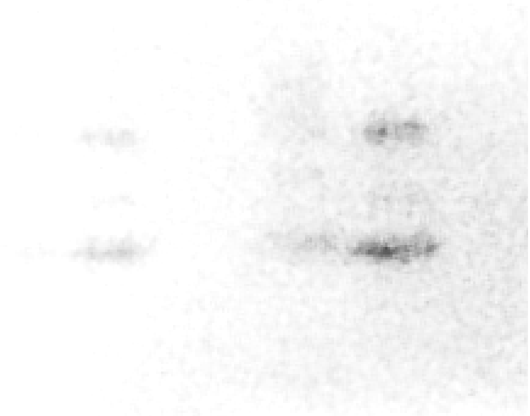

PSN1 MIAPaCa2
DMSO/C646 30uM, 48 hours Cl Cas3

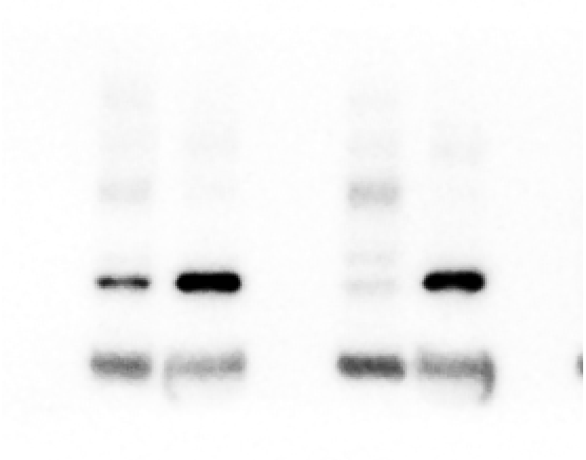

PSN1 MIAPaCa2
DMSO/C646 30uM, 48 hours Cl PARP

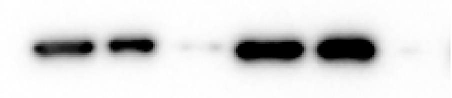

PSN1 MIAPaCa2
DMSO/C646 30uM, 48 hours β-actin
